# Spatiotemporal Patterns of Small for Gestational Age and Low Birth Weight Births and Associations With Land Use and Socioeconomic Status

**DOI:** 10.1177/1178630219869922

**Published:** 2019-08-22

**Authors:** Charlene C Nielsen, Carl G Amrhein, Prakesh S Shah, Khalid Aziz, Alvaro R Osornio-Vargas

**Affiliations:** 1 Department of Pediatrics, University of Alberta, Edmonton, AB, Canada; 2 Department of Earth and Atmospheric Sciences, University of Alberta, Edmonton, AB, Canada; 3 Faculty of Arts and Sciences, The Aga Khan University, Nairobi, Kenya; 4 Faculty of Arts and Sciences, The Aga Khan University, Karachi, Pakistan; 5 Department of Pediatrics and Institute of Health Policy, Management, and Evaluation, University of Toronto, Mount Sinai Hospital, The Canadian Neonatal Network, Toronto, ON, Canada

**Keywords:** Small for gestational age, low birth weight at term, environmental health, socioeconomic status, space-time pattern mining, exposome

## Abstract

In addition to small for gestational age (SGA) and low birth weight at term (LBWT), critically ill cases of SGA/LBWT are significant events from outcomes and economic perspectives that require further understanding of risk factors. We aimed to assess the spatiotemporal distribution of locations where there were consistently higher numbers of critically ill SGA/LBWT (hot spots) in comparison with all SGA/LBWT and all births. We focused on Edmonton (2008-2010) and Calgary (2006-2010), Alberta, and used a geographical information system to apply emerging hot spot analysis, as a new approach for understanding SGA, LBWT, and the critically ill counterparts (ciSGA or ciLBWT). We also compared the resulting aggregated categorical patterns with proportions of land use and socioeconomic status (SES) using Spearman correlation and logistic regression. There was an overall increasing trend in all space-time clusters. Whole period emerging hot spot patterns among births and SGA generally coincided, but SGA with ciSGA and LBWT with ciLBWT did not. Regression coefficients were highest for low SES with SGA and LBWT, but not with ciSGA and ciLBWT. Open areas and industrial land use were most associated with ciLBWT but not with ciSGA, SGA, or LBWT. Differences in the space-time hot spot patterns and the associations with ciSGA and ciLBWT indicate further need to research the interplay of maternal and environmental influences. We demonstrated the novel application of emerging hot spot analysis for small newborns and spatially related them to the surrounding environment.

## Background

Being born too small, such as low birth weight at term (LBWT)—defined as birth weight below 2500 g for full-term pregnancy—is considered an adverse birth outcome because it is associated with infant mortality, physical and cognitive disabilities, and long-term health issues.^1-3^ However, this absolute parameter does not take into consideration gestational age. To account for variability in birth weight at different gestations, another parameter called small for gestational age (SGA) is used. Small for gestational age is defined as birth weight below the 10th centile weight, based on sex and weeks of gestation.^4^

In Canada, the average rate of SGA was reported to be 9.1% and low birth weight (LBW; all gestational ages < 2500 g) was 6.4%, during 2015 to 2017,^5^ whereas in Alberta, the rate of SGA was 10.1% and LBW was 7.1%. Refer to supplemental Figure S1 to see how these values have been increasing since before the beginning of our study. Disorders related to short gestation and LBW are the second leading cause of infant death in Canada.^6^ Both these outcomes are associated with adverse consequences with higher rates of admission to neonatal intensive care units (NICUs), resulting in higher economic and social costs.^2,7^ Newborns admitted to NICUs—and who are also SGA and/or LBWT (ie, 37 or more weeks gestation)—are considered critically ill (ci); ie, ciSGA and ciLBWT.

Maternal conditions (eg, preexisting and pregnancy-related health conditions, behavior, and nutrition) are important risk factors for SGA/LBWT,^8-11^ but they do not fully explain the occurrence. The role of environmental factors in causation of SGA/LBWT has been suspected; however, no firm conclusion/attribution has been delineated in previous studies.^12-15^ To reveal patterns and associations between SGA/LBWT and the environment that may not be evident in traditional spatial epidemiology, spatial statistics and geographic data mining in geographical information system (GIS) allow for spatial-temporal variation because interactions of the environment are not constant.^16^ Geographical information systems are valuable for understanding patterns and the differences among births and SGA/LBWT because GIS provide various mapping techniques for public health data.^17-19^ Using GIS to also analyze spatiotemporal patterns has the potential to identify priority areas for management and intervention, as has been established in other space-time pattern studies in health, crime, and conservation.^20-23^ Kirby et al^24^ described common spatiotemporal clustering methods used to detect hot spots, which may be defined as “unusual concentrations of health events in space and time.”^17^ A natural application for spatiotemporal analysis are birth events,^25^ and one such study by Ozdenerol et al found various methods generated vastly differing, but somewhat complementary, results from the same individual data. Here, we apply the newer emerging hot spot analysis (EHSA), which has not previously been applied to any birth outcomes, including SGA/LBWT.

Thus, our objective was to examine how hot spot patterns—in space and time—compare among pregnancies that resulted in SGA/LBWT and those that resulted in ciSGA/ciLBWT. In addition, and in an effort to further understanding of the exposome (ie, the measure of all the exposures of an individual in a lifetime and how those exposures relate to health), we aimed to understand where the patterns coincide with the surrounding environment, specifically land use and area-level socioeconomic status (SES).

## Methods

### Study design and setting

We conducted our retrospective study between the years 2006 and 2010 inclusive using Canadian Neonatal Network (CNN) and Alberta Perinatal Health Program (APHP) databases.

The CNN maintains a standardized NICU database that included all admissions to NICUs in 19 urban centers in Canada.^26^ The database has shown a very high internal consistency and reliability.^27^ The APHP databases included all births, whereas the CNN database included critically ill births (which were also included in APHP database), which allowed us to compare patterns of all SGA/LBWT births with patterns of critically ill SGA/LBWT births. Due to the restriction of on-site access to each database, these databases were not linked; however, the resulting space-time hot spot patterns can be compared between the 2 groups of neonates.

**Figure 1.**
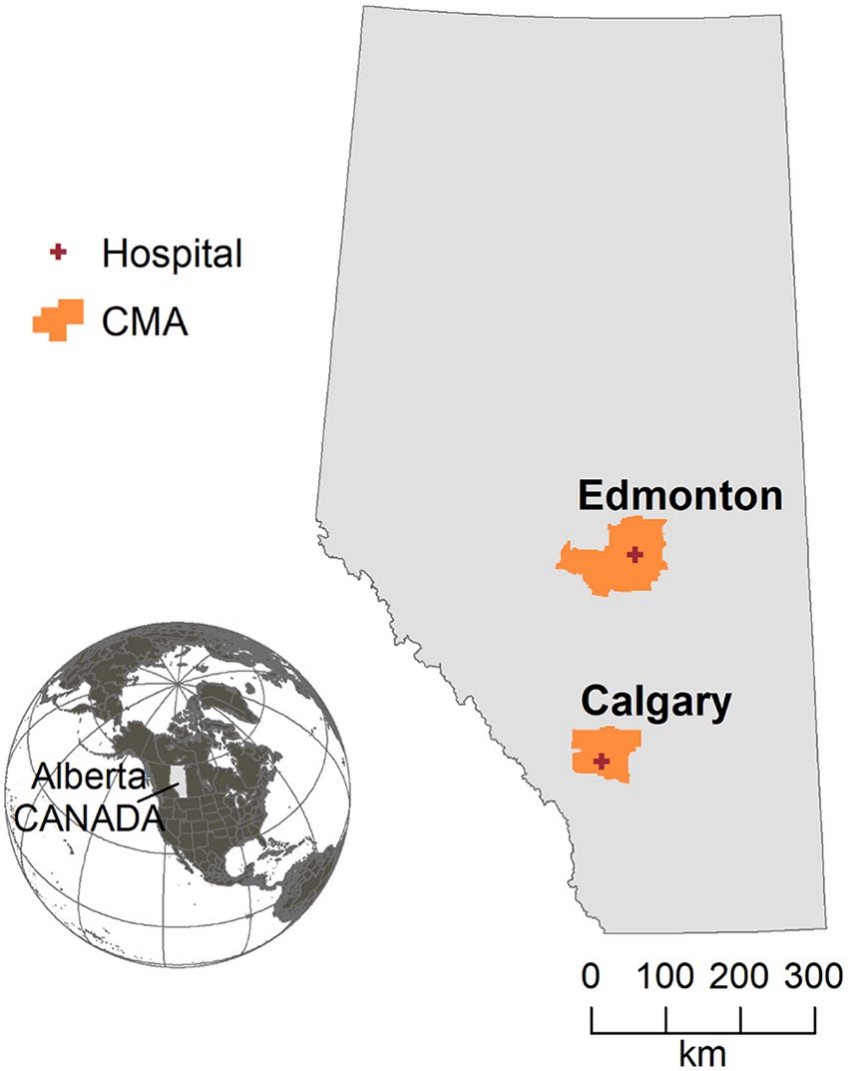
The study focused on the Calgary and Edmonton Census Metropolitan Areas (CMA), in the province of Alberta, Canada, served by hospitals with neonatal intensive care units participating in the Canadian Neonatal Network.

We defined the primary areas served by the CNN NICUs as census metropolitan areas (CMAs). A CMA is essentially urban core and surrounding municipalities integrated by commuting flows and having a minimum total population of 100 000.^28^ According to census geography hierarchy, a CMA is composed of contiguous census subdivisions that may cross census division and provincial boundaries. Our study area involved the Calgary and Edmonton CMAs, shown in Figure 1, and described in Table 1 in terms of size and population.

**Table 1.** Census Metropolitan Area (CMA) characteristics from the 2011 Census for Canada.

CMA	Area (km^2^)	Population		
		Total	Women: 15 to 44 y	Infants: 0 to 4 y
Calgary	5108	1 214 839	272 320	80 855
Edmonton	9427	1 159 869	252 085	73 645

The APHP is an administrative clinical registry that collects and standardizes demographic information on all hospital births and out of hospital births (attended by registered midwives) for the province of Alberta.^29^ The provincial data were subset to the 2 CMAs to compare with the CNN data. Calgary had 5 years (2006-2010) of CNN data, but Edmonton had 3 years because the participating hospital did not join the CNN until 2008.

**Figure 2.**
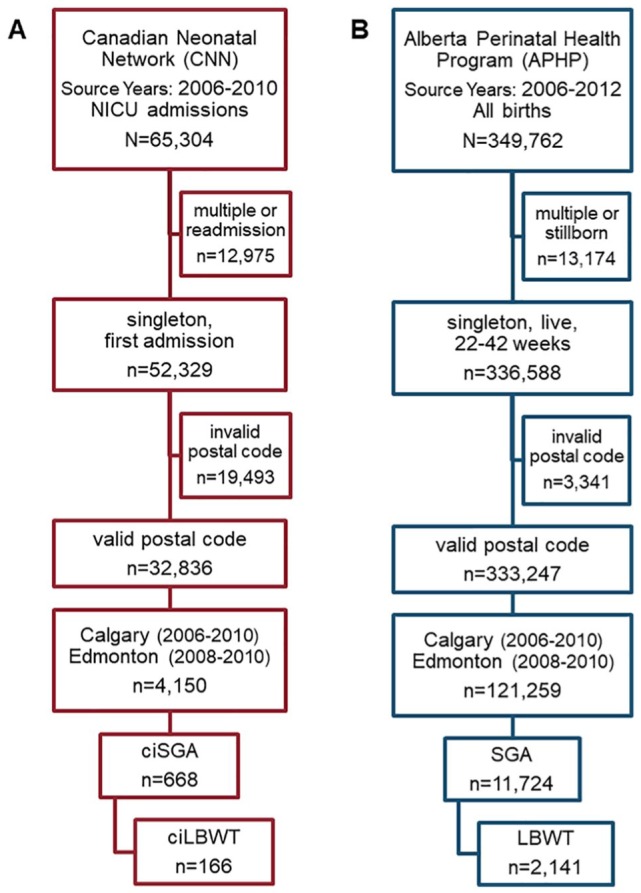
The birth locations from (A) Canadian Neonatal Network (CNN) and (B) Alberta Perinatal Health Program (APHP) data were subset to valid postal codes within the extent of Census Metropolitan Areas (CMAs): Calgary (2006-2010) and Edmonton (2008-2010). ciLBWT indicates critically ill low birth weight at term; ciSGA, critically ill small for gestational age; LBWT, low birth weight at term; NICU, neonatal intensive care unit; SGA, small for gestational age.

Both CNN and APHP provided anonymized records of birth weight (grams), gestational age (completed weeks), sex, single/multiple, admission status (CNN only), pregnancy outcome (APHP only), and the residential postal code. As depicted in Figure 2, we selected singletons at first admission (CNN) and live births (APHP) with valid postal codes. The large reduction of records in the CNN database was due primarily to our initial selection criteria of only including postal codes located inside each CMA.

### Dependent variables

Outcomes of interest were LBWT, defined as birth weight below 2500 g at weeks 37 to 42, and SGA, defined as birth weight below the 10th centile for gestational age and sex according to Canadian reference values.^4^ Small for gestational age and LBWT were from the APHP database. The critically ill (ci)—ciSGA or ciLBWT—were classified as those SGA and LBWT neonates who were also admitted to the NICU and were from the CNN database.

### Independent variables

To help understand the SGA/LBWT patterns, we examined their relationships with landscape-level variables relevant to birth outcomes. These included the surrounding land use and the area-level SES.

Digital Mapping Technologies Inc. (DMTI) Spatial provided a land use classification for the urban areas across Canada.^30^ We grouped the 7 standardized patterns of construction and activity that land was used for into 4 general categories: *services* (commercial, government/institution), *open areas* (open area, parks and recreation, waterbody), *residential*, and *industry* (resource and industry). Due to linkage with environmental pollutants, the primary category of interest was industry, defined as land occupied by establishments engaged in the mechanical or chemical transformation of materials or substances into new products or land set aside for the extraction or production of renewable and nonrenewable resources. The land use categories are mapped for Calgary in Supplemental Figure S2A and Edmonton in Supplemental Figure S3A.

Chan et al^31^ provided a comprehensive index of Canadian SES that is suitable for research in health and environmental pollutants. The area-level SES index was developed from the 2006 Census Canada by incorporating 22 variables on culture, potential existence of indoor environmental pollutants, environmental injustice indicators, and deprivation variables in a principal components analysis for each dissemination area (DA). A DA was the smallest, relatively stable, geographic unit within which all census data were distributed and was composed of contiguous dissemination blocks having a total population of 400 to 700.^28^ We grouped the SES reported as quintile values into the following levels—*low* (1 and 2), *medium* (3 and 4), and *high* (5)—to indicate relative SES for the DA. The SES levels are mapped for Calgary in Supplemental Figure S2B and Edmonton in Supplemental Figure S3B.

### Geolocation

In a process called geolocation, we assigned the latitude and longitude coordinates to the CNN and APHP records by joining the 6-character postal codes to DMTI Spatial’s Platinum Postal Code Suite database.^32^ This database consists of population-weighted centroids of the postal code delivery unit. To ensure static locations throughout the study period, we uniquely selected postal codes from 2001 through 2013 (the time span was necessary due to addition of new postal codes and retirement of old ones).

**Figure 3.**
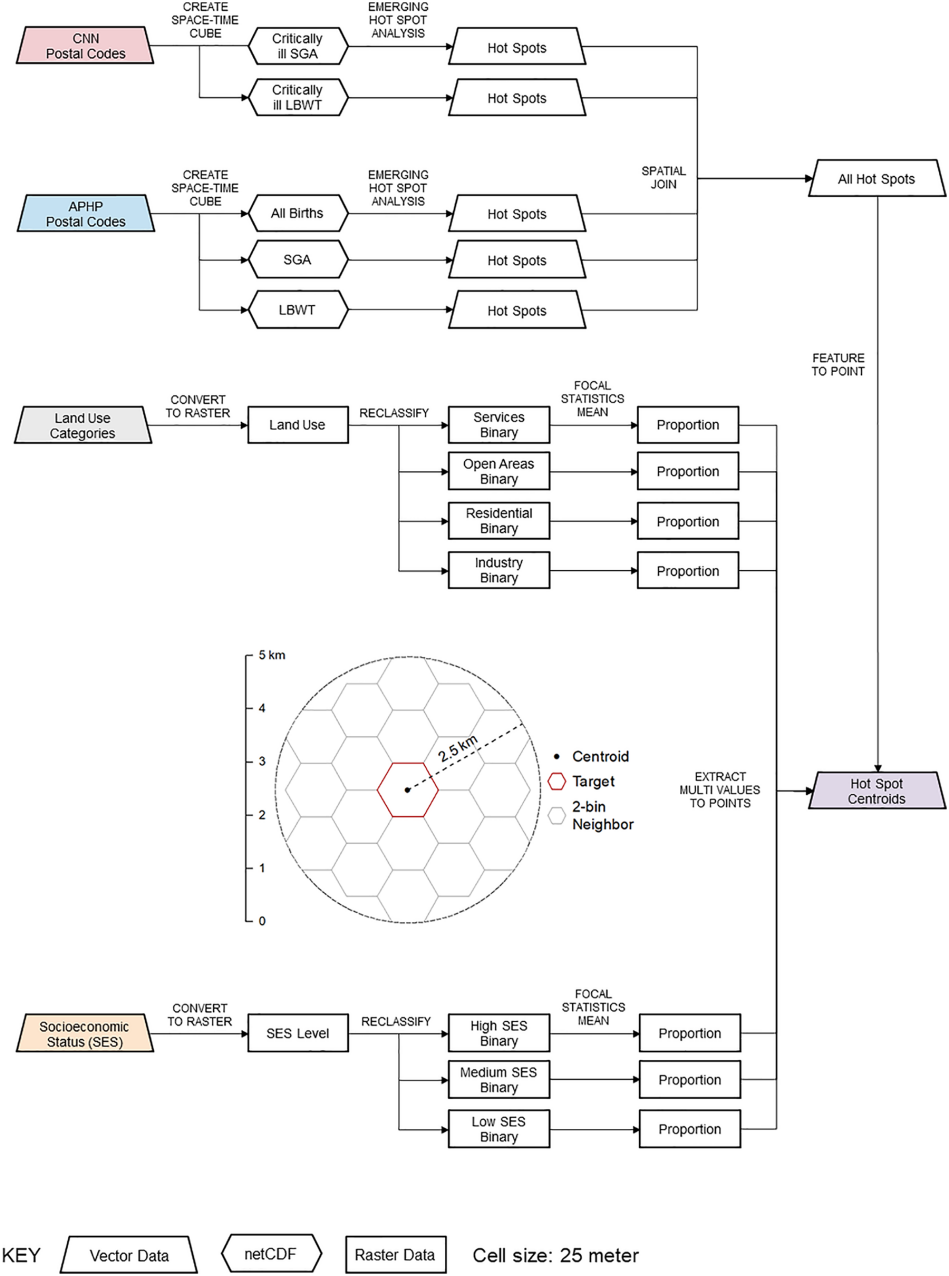
Flow chart of GIS commands for analyzing small newborns in space and time. APHP indicates Alberta Perinatal Health Program; CNN, Canadian Neonatal Network; GIS, geographical information system; LBWT, low birth weight at term; netCDF, network Common Data Form; SES, socioeconomic status; SGA, small for gestational age.

Figure 3 shows the analytical steps that are described in the sections below. We used Esri’s ArcGIS Desktop 10.6^33^ and Pro 2.0^34^ software.

### Spatial-temporal patterns

We analyzed the distributions and patterns of each SGA/LBWT and all births—for both the CNN and APHP data—in the context of both space and time using the ArcGIS space-time pattern mining tools.^35^ For each CMA, we transformed the postal codes time-stamped by birthdate into multidimensional data cubes, stored as network Common Data Form (netCDF) files, by (1) aggregating the points—spatially in 1-km-high hexagon bins and temporally in 1-month time slices, (2) summing the binary values of SGA or LBWT, (3) filling empty bins with zeros, and (4) aligning to a reference time equal to the beginning of the study (January 1, 2006 for Calgary and January 1, 2008 for the Edmonton CMA). The Mann-Kendall statistic evaluated the trend in SGA/LBWT point counts for each data cube.

The hexagon was chosen because it is more natural in shape, better represents connectivity, and minimizes edge effects^36^; the 1-km size fit within typical city neighborhoods and helped protect individual privacy. The 1-month time-step interval fit within a trimester. Bins were filled with zeros because SGA and LBWT are considered rare events, counted in whole numbers, and therefore interpolation would not be appropriate. The reference time ensured all SGA/LBWT would have the same start date for comparison purposes. On average, 32 postal codes were aggregated into 1-km hexagons, with a mean size of 0.866 km^2^ or 86.6 ha.

Emerging hot spot analysis analyzed each data cube by calculating statistically significant hot and cold spot trends in SGA and LBWT using 2 statistics. The Getis-Ord Gi* statistic assessed the location and degree of spatial clustering by calculating the *z* score, *P* value, and hot spot bin classification. The Mann-Kendall statistic evaluated these measures to assess temporal trends and then categorized locations according to Supplemental Table S1. The interested reader may refer to Esri^35^ and Harris et al^23^ for fuller details on the spatiotemporal statistics and the standard categories resulting from EHSA.

To simulate city neighborhood sizes, we used a fixed distance of 2001 m (note: the additional 1 m ensured that complete hexagons were included), which encompassed the current hexagon and 2 adjacent hexagons (2.5-3 km). To simulate a trimester, we used 2 time steps, which included the current month and previous 2 months (3 months). Hot spot maps were output to visualize the spatial-temporal significance of SGA, LBWT, and all births (from APHP only) in each CMA for the study period.

### Neighborhood proportions

For both the independent variables, we reclassified the categorical values (land use, n = 4; SES, n = 3) into separate binary surfaces, where “1” indicated presence and “0” indicated absence. Then, we applied a neighborhood moving-window analysis, called focal statistics. Calculating the mean statistic within a 2500-m radius on the binary surfaces resulted in proportions. We assigned the proportions of land use and SES to the centroids of the hexagons that resulted from the EHSA for each SGA/LBWT. The 2500-m neighborhood estimated the proportions of each land use or SES class within the distance defined for the EHSA described above.

### Statistical analyses

For each CMA, we spatially joined all hot/cold spots maps, calculated Spearman correlation on the pattern categories ranked from coldest to hottest, and used the resulting statistics to determine the association of (1) SGA/LBWT with all births or (2) critically ill cases with all SGA/LBWT of the same type. The categories were also correlated with the land use and SES proportions to help determine any relationships with SGA/LBWT.

To explore the relationship of each SGA/LBWT hot spots and surrounding proportions of land use and SES, we used logistic regression. Binary variables were coded as “1” for all hot spot categories and as “0” for non–hot spot categories. Because the land use and SES categories were each mutually exclusive proportions, we specified residential and high SES as the reference categories to test our hypothesis that the target categories of industry and low SES have the highest associations with SGA/LBWT hot spot patterns, if no collinearity exists. To account for areas having more births, we included the covariate sum of births (from APHP data) in each hexagon bin over the entire study period. We used STATA 12 statistical software.^37^ Because we were interested only in the significance of the effect of 1 independent variable (*X*) on the response (*Y*), and the data were not appropriate for implying risk, only the coefficients were calculated (ie, logarithm of the odds ratios), along with the 95% confidence intervals (CIs) and *P* values. We used the magnitude of the coefficient, whether the CIs were on the same side of 0 as the coefficient, and *P* values < .05 to identify the stronger associations.

## Results

### Characteristics of the study population

The 2 CMAs varied in the raw counts of all births, all small newborns (SGA or LBWT), and critically ill small newborns. As shown in Table 2, Calgary had 77 711 total births over 5 years; there were 7907 (10.2%) SGA, 505 (0.7%) ciSGA, 1462 (1.9%) LBWT, and 126 (0.2%) ciLBWT. For Edmonton’s 43 548 births over 3 years, there were 3817 (8.8%) SGA, 163 (0.4%) ciSGA, 679 (1.6%) LBWT, and 40 (0.1%) ciLBWT.

**Table 2.** Census Metropolitan Area (CMA) number of records from the Alberta Perinatal Health Program (APHP) and Canadian Neonatal Network (CNN) databases for only the records having valid 6-character postal codes.

CMA	Years	APHP			CNN		
		Births	SGA	LBWT	NICU admissions	ciSGA	ciLBWT
Calgary	2006-2010	77 711	7907	1462	2908	505	126
Edmonton	2008-2010	43 548	3817	679	1242	163	40
Both CMAs		121 259	11 724	2141	4150	668	166

Abbreviations: ciLBWT, critically ill low birth weight at term; ciSGA, critically ill small for gestational age; LBWT, low birth weight at term; NICU, neonatal intensive care unit; SGA, small for gestational age.

Edmonton did not report all admissions >33 weeks gestation.

### Space-time cube trends

When the space-time cubes were created, information on the overall data trend was reported. The nonparametric Mann-Kendall statistic, an aspatial time-series analysis, indicated whether the events increased or decreased over time by evaluating count values for the locations in each 3-month time-step interval for our study. Table 3 contains the trend statistics, which showed increasing trends for every SGA/LBWT and births, in both CMAs. The Mann-Kendall statistics ranged from 1.86 to 4.89 (*P* values: <.01-.06) in Calgary and 2.56 to 6.72 (*P* values: <.01-.01) in Edmonton; both were positive and much higher than the expected zero value if there was no trend.

**Table 3.** Space-time cubes and emerging hot spot analyses exhibiting increasing trends across Alberta Perinatal Health Program (APHP) all births, small for gestational age (SGA), low birth weight at term (LBWT) and Canadian Neonatal Network (CNN) critically ill (ci) SGA and LBWT.

	Calgary					Edmonton				
	APHP = 865 locations			CNN = 568 locations		APHP = 1032 locations			CNN = 442 locations	
	Births	SGA	LBWT	ciSGA	ciLBWT	Births	SGA	LBWT	ciSGA	ciLBWT
Trend	↑	↑	↑	↑	↑	↑	↑	↑	↑	↑
Mann-Kendall statistic	4.89	3.07	1.86	3.65	2.22	6.72	6.66	5.72	3.71	2.56
*P* value	<.01	<.01	.06	<.01	.03	<.01	<.01	<.01	<.01	.01
Sparseness (% non-zero)	52.75	12.8	2.70	1.46	0.36	27.57	5.38	1.07	0.56	0.14
No pattern	0.508	0.874	0.939	0.979	0.944	0.421	0.684	0.898	0.937	0.939
Hot spots										
New	−	0.001	0.010	0.002	0.018	−	0.008	0.004	0.014	−
Consecutive	0.003	−	−	0.004	0.018	0.002	0.045	0.002	0.011	0.009
Intensifying	0.112	0.015	−	−	−	−	−	−	−	−
Persistent	0.045	0.020	−	−	−	−	−	−	−	−
Diminishing	0.013	0.003	−	−	−	−	−	−	−	−
Sporadic	0.082	0.084	0.051	0.016	0.021	0.009	0.264	0.096	0.038	0.052
Oscillating	0.006	−	−	−	−	0.513	−	−	−	−
Historical	0.001	0.001	−	−	−	−	−	−	−	−
Cold spots										
New	0.001	−	−	−	−	−	−	−	−	−
Consecutive	−	−	−	−	−	−	−	−	−	−
Intensifying	0.043	−	−	−	−	−	−	−	−	−
Persistent	0.090	−	−	−	−	−	−	−	−	−
Diminishing	0.014	−	−	−	−	0.016	−	−	−	−
Sporadic	0.082	−	−	−	−	0.040	−	−	−	−
Oscillating	−	−	−	−	−	−	−	−	−	−
Historical	−	−	−	−	−	−	−	−	−	−
Hot/cold trends	0.492	0.126	0.061	0.021	0.056	0.579	0.316	0.102	0.063	0.061
Category count	12	6	2	3	3	5	3	3	3	2

Proportion of each hot/cold spot category is shown; pattern categories are defined in Supplemental Table S1.

### Emerging hot spot patterns

The space-time analyses occurred within a 3-dimensional model, but the results were multiple categories, explained in Supplemental Table S1, and are only suitable for representation in 2-dimensional maps. Table 3 identifies the patterns that resulted from the EHSA for each SGA/LBWT in the CMAs. Because the areal and temporal extents differed in each study area, the proportions of each category are shown. The EHSA pattern categories are defined in Supplemental Table S1 within the context of Calgary’s 60-month and Edmonton’s 36-month time series. Calgary had more variability in hot/cold spots with 2 to 12 categories; Edmonton had 2 to 5 categories. The largest proportions of both CMAs had no patterns. Small amounts of new hot spots were present for SGA/LBWT and ciSGA, but none for Edmonton’s ciLBWT. Consecutive hot spots occurred in all SGA/LBWT for Edmonton, but only for ciSGA/ciLBWT and all births in Calgary. Intensifying, persistent, and diminishing hot spots occurred in Calgary for all births and SGA. Sporadic hot spots were present in all births and every SGA/LBWT, with the highest proportion in Edmonton’s SGA. Oscillating hot spots had the highest proportion in Edmonton but occurred in both CMAs for all births. Cold spots occurred in both CMAs (Calgary had 6 cold categories; Edmonton had 2), but only for all births. Overall, the proportions of each pattern indicated that sporadic and consecutive hot spots dominated the trends, and births in both CMAs also exhibited cold spots.

### Pattern comparisons among SGA/LBWT

**Figure 4.**
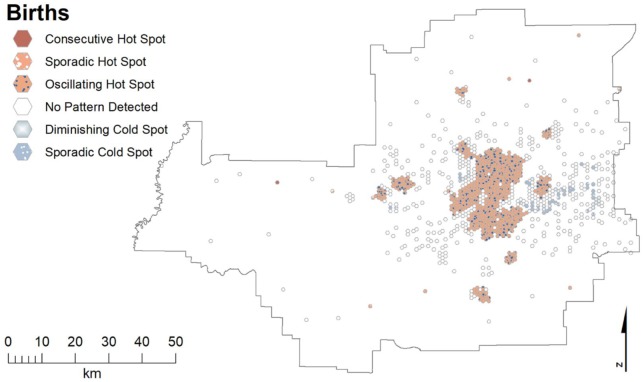
Emerging hot spots of all births in the Edmonton CMA. CMA indicates census metropolitan areas.

**Figure 5.**
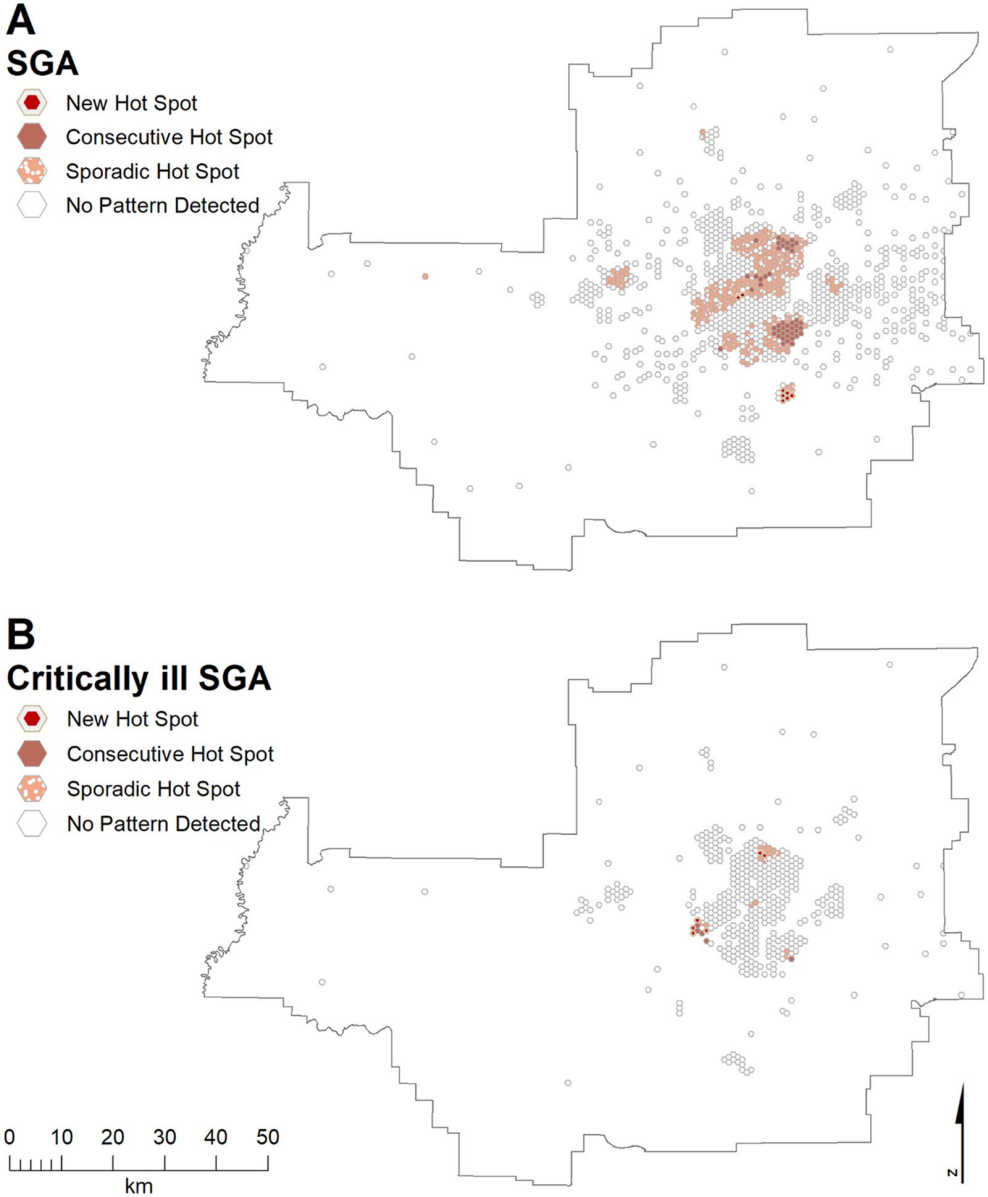
Emerging hot spots of (A) SGA and (B) critically ill SGA in the Edmonton CMA. CMA indicates census metropolitan areas; SGA, small for gestational age.

**Figure 6.**
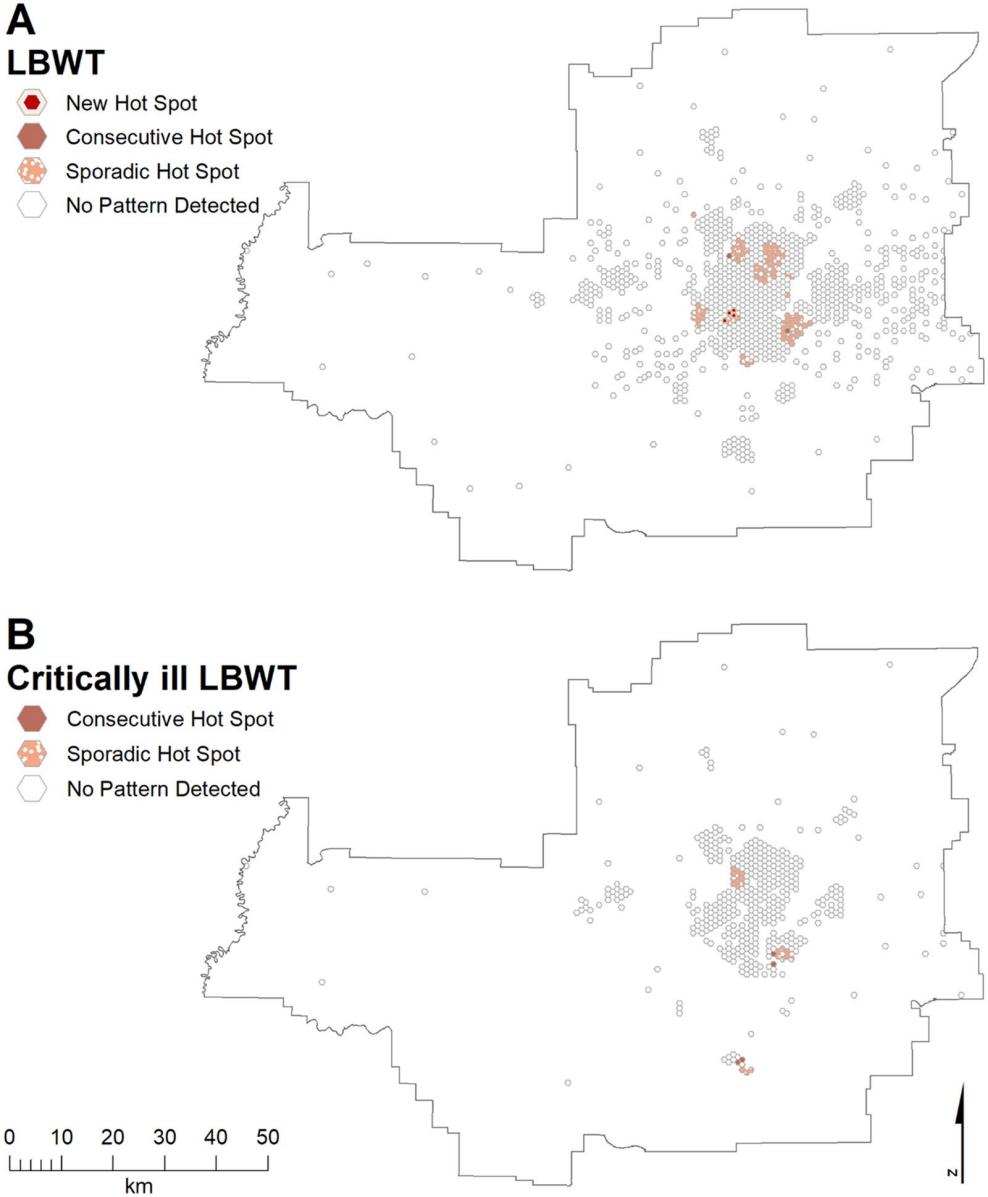
Emerging hot spots of (A) LBWT and (B) critically ill LBWT SGA in the Edmonton CMA. CMA indicates census metropolitan areas; LBWT, low birth weight at term; SGA, small for gestational age.

In Edmonton, there were oscillating hot spots for all births covering most of the core CMA (Figure 4). Figure 5A shows distinct areas of SGA occurred in a large band from the northeast through central to west, across the south, and in outlying communities. Much smaller areas were seen for ciSGA: north-central, west, and southeast (Figure 5B). Figure 6A shows hot spots for LBWT in the north-northwest, north-central, southeast, west of central, west, and south. Three distinct areas were seen for ciLBWT: northwest, south-southeast, and an outlying community (Figure 6B).

Refer to the supplemental material to see the hot spot patterns in Calgary (Supplemental Figures S4-S8). Enlargements of Figures 4 through 6 of the Edmonton CMA are also available in the supplemental material (Supplemental Figures S9-S13).

Table 4 reports the Spearman correlations among all births, SGA/LBWT, and ciSGA/ciLBWT. For both CMAs, the associations ranged from ρ 0.09 to 0.48, *P* < .05, with the highest between all births-SGA. The correlations decreased from SGA/LBWT to ciSGA/ciLBWT (*P* < .05): in Edmonton, all births-SGA was ρ = 0.48, SGA-ciSGA was ρ = 0.18, all births-LBWT was ρ = 0.18, and LBWT-ciLBWT was ρ = 0.13; similar correlations were seen in Calgary.

**Table 4.** Spearman correlation (ρ) statistics comparing emerging hot spot patterns for all births, SGA/LBWT, and critically ill (ci) SGA/LBWT by Census Metropolitan Area (CMA).

Spearman ρ	Edmonton					Calgary				
	Births	SGA	LBWT	ciSGA	ciLBWT	Births	SGA	LBWT	ciSGA	ciLBWT
Births	1					1				
SGA	0.48*	1				0.47*	1			
LBWT	0.18*	0.23*	1			0.31*	0.47*	1		
ciSGA	0.10*	0.20*	0.19*	1		0.09*	−0.03	0.08	1	
ciLBWT	0.12*	−0.13*	0.13*	0.09	1	0.17*	−0.01	0.15*	0.23*	1

Abbreviations: ciLBWT, critically ill low birth weight at term; ciSGA, critically ill small for gestational age; LBWT, low birth weight at term; SGA, small for gestational age.

Significant ρ values (*P* < .05) are marked with an asterisk (*).

### Associations of space-time patterns with land use and SES

The direction and relative rho values of Spearman correlations gave insight to which land use and SES categories had any relationships with the SGA/LBWT space-time hot spot patterns. As shown in Supplemental Table S2, all births and SGA were associated the most with land use and SES categories for ρ > |0.4.|

In Edmonton, SGA hot spots were positively associated with low SES (ρ = 0.43), residential land use (ρ = 0.44), and negatively with open areas (ρ = –0.40) but were also negatively associated with high SES (ρ = –0.41); no strong associations were seen for LBWT or either ciSGA/ciLBWT.

In Calgary, SGA hot spots were negatively associated with high SES (ρ = –0.42); no strong associations were seen for all births, LBWT, or either ciSGA/ciLBWT.

Supplemental Table S3 indicates the correlation between land use and area-level SES, suggesting the variables of interest were relatively less independent in the Edmonton CMA, but independent in the Calgary CMA. Open areas and services were noticeably negatively correlated (Edmonton ρ = –0.73; Calgary ρ = –0.66), and the same negative relationship was seen for open areas and residential (Edmonton ρ = –0.84; Calgary ρ = –0.85).

The logistic regression model coefficients are displayed in Table 5, where residential land use and high SES were the reference variables. According to the pseudo *R*^2^ values, the model fit ranged from 0.30 (ciSGA, Edmonton) to 0.45 (SGA, Calgary and Edmonton), meaning 30% to 45% of the SGA/LBWT hot spot variations were explained by area-level land use and SES.

**Table 5.** Logistic regression β coefficients (and 95% CI) for all SGA/LBWT and ciSGA/ciLBWT modeled with proportions of surrounding land use categories and level of socioeconomic status (SES).

	SGA	LBWT	ciSGA	ciLBWT
Edmonton model β coefficient (95% CI)				
Services	−30.1 (−40.2, −20.1)*	−34.9 (−47.3, −22.4)*	−15.2 (−25.3, −5.1)*	−13.5 (−23.8, −3.1)*
Open areas	−7.0 (−8.4, −5.5)*	−4.2 (−5.9, −2.6)*	1.6 (0.5, 2.7)*	1.6 (0.5, 2.8)*
Industry	−5.7 (−7.5, −3.9)*	−6.1 (−8.7, −3.6)*	1.1 (−0.7, 2.9)	2.3 (0.4, 4.2)*
SES low	3.4 (2.4, 4.4)*	4.5 (3.2, 5.7)*	0.6 (−0.3, 1.6)	0.5 (−0.4, 1.5)
SES medium	3.3 (2.4, 4.3)*	0.9 (−0.4, 2.2)	−0.3 (−0.9, 0.4)	−0.6 (−1.3, 0.1)
Sum births	0.01 (0.01, 0.01)*	−0.03 (−0.03, −0.02)*	0.00 (0.00, 0.01)	−0.03 (−0.04, −0.03)*
Intercept	1.2 (0.1, 2.2)*	0.88 (−0.13, 1.89)	−0.7 (−2.3, 0.8)	1.0 (0.0, 2.0)
LR χ^2^	579.5	494.2	203.6	537.2
Pseudo *R*^2^	0.45	0.36	0.30	0.39
Calgary model β coefficient (95% CI)				
Services	5.8 (−17.0, 28.6)	4.5 (−21.5, 30.6)	−18.6 (−37.8, 0.7)	−7.5 (−23.4, 8.5)
Open areas	−1.4 (−3.8, 1.0)	−0.4 (−3.0, 2.1)	0.9 (−0.3, 2.2)	1.7 (0.6, 2.8)*
Industry	2.3 (−0.2, 4.7)	−3.5 (−7.5, 0.6)	0.8 (−1.3, 2.9)	3.4 (1.6, 5.2)*
SES low	4.9 (3.7, 6.2)*	3.9 (2.5, 5.4)*	0.8 (−0.1, 1.8)	0.1 (−0.7, 0.9)
SES medium	1.4 (−0.2, 3.0)	1.1 (−1.0, 3.2)	0.2 (−0.6, 1.0)	−0.4 (−1.1, 0.3)
Sum births	0.01 (0.01, 0.01)*	−0.04 (−0.04, −0.03)*	0.01 (0.00, 0.01)*	−0.02 (−0.02, −0.02)*
Intercept	−5.4 (−7.4, −3.4)*	0.5 (−0.7, 1.7)	−5.1 (−7.3, −2.8)*	−0.3 (−1.3, 0.7)
LR χ^2^	294.5	503.1	129.3	368.2
Pseudo *R*^2^	0.45	0.45	0.32	0.32

Abbreviations: ciLBWT, critically ill low birth weight at term; ciSGA, critically ill small for gestational age; CI, confidence interval; LBWT, low birth weight at term; LR, likelihood ratio; SGA, small for gestational age.

Residential and high SES were the reference categories; LR χ^2^ significance is *P* < .001; significant coefficients (*P* < .05) are marked by an asterisk (*); number of locations are indicated in Table 3.

In Edmonton (*P* < .05), SGA hot spots were surrounded by low SES (β = 3.4 [95% CI: 2.4, 4.4]) and medium SES (β = 3.3 [95% CI: 2.4, 4.3]), LBWT hot spots were surrounded by low SES (β = 4.5 [95% CI: 3.2, 5.7]), ciSGA hot spots had slightly more open areas (β = 1.6 [95% CI: 0.5, 2.7]), and ciLBWT hot spots had more industry (β = 2.3 [95% CI: 0.4, 4.2]) and open areas (β = 1.6 [95% CI: 0.5, 2.8]). Due to high correlation of most land use variables with low SES (Supplemental Table S2), we calculated the variance inflation factors (VIFs: Supplemental Table S4). According to the VIF <10 threshold indicated by Chatterjee and Hadi,^38^ our VIFs ⩽4.19 suggest that collinearity among SES and land use was not problematic. In Supplemental Table S5, we show the β coefficients from logistic regression analyses of only SES in Edmonton and only SES and industrial land use in Calgary adjusted by total births. When land cover variables were removed from the model and only SES remained, the coefficients for SES were relatively stable (Supplemental Table S5). This illustrates that inferences on SES were robust regardless of inclusion of land use variables.

In Calgary, the associations were the same as seen in Edmonton with the exception that the ciSGA hot spots were not significantly different from the reference.

## Discussion

Hot spots for ciSGA and ciLBWT occurred in different locations than all SGA/LBWT, but hot spots of both SGA and LBWT logically occurred in the same locations as hot spots for all births. The differing locations were counterintuitive for the critically ill hot spots, suggesting there may be neighborhood-level environmental influences unevenly distributed across the cities or other unmeasured variables in play.

The increasing trends of SGA/LBWT in each CMA were supported by increasing trends of all births: SGA/LBWT hot spot space-time clusters were increasing because birth hot spots were increasing. However, the locations did not coincide across the study areas, and the relatively low correlation values (ie, ρ 0.10 to <0.30)^39^ with the critically ill quantified this difference in hot spot patterns. If the critically ill hot spots were in the same locations as SGA/LBWT, then there may be homogeneous risk factors for both conditions at those locations. We suspect that different aspects of the exposome may be participating differently and more strongly for critically ill and SGA/LBWT in different locations for these multifactorial health conditions.

The regression coefficients supported that low SES and industrial land use had the highest associations, depending on the birth outcome. Although similar spatial associations with low SES have been reported before,^40-42^ the association with land use has received less attention. The low regression coefficients for the ciSGA/ciLBWT suggest that maternal factors and/or other environmental exposures, such as urban air pollutants, may be additionally important for these types of cases.^15,43,44^ Higher amounts of surrounding open spaces were associated with ciSGA and ciLBWT hot spots, implying that there may be less access to health services and supported by the negative correlations of open spaces with services, as others have also suggested.^40,42^ The opposite associations were seen between all and critically ill newborns: land use was not significant with all small newborns, and SES was not significant with the critically ill.

In Canada, there is a paucity of published studies on the spatial and temporal trends of SGA/LBWT, especially for the critically ill small newborns. Statistics Canada has reported that small newborns are increasing over time for our geographical areas of interest.^5^ Nielsen et al^45^ published on the spatial distribution of SGA and LBWT for the entire province but comparisons cannot be made due to methodological differences. As for ciSGA/ciLBWT, there are no published temporal trends for each city participating in the CNN to compare to. The space-time patterns demonstrated here agree with the increasing national trend, but additionally pinpoint the locations of where there are hot spots of concern.

Although we had access to all records from the APHP and CNN databases, the postal code locations may not have been as accurate for the less urban areas in each CMA. Similarly, the SES index outside of urban areas did not have as accurate spatial resolution because the DAs may be vast. Larger areas are encompassed by the postal delivery units and DAs in rural areas.

The CNN data collection methods differed between the 2 CMAs, where Edmonton did not report critically ill newborns having gestational ages >33 weeks unless they were admitted to the surgical unit. Although the results appear to be similar to the Calgary CMA, the data reporting and year of participation difference mean direct comparisons cannot be made between the CMAs. This study was not hospital-specific, meaning that the analysis was based on the maternal residential postal code and may include a miniscule number of NICU admissions to hospitals not in the same CMA as the residences. This also meant that critically ill births from mothers living in the CMA may have been reported at another facility and therefore not captured in the CNN database.

Although the reporting of coefficients (log of odds ratios) from the logistic regression model may not be suitable for alternative objectives (eg, in epidemiology or planning policy), the beta coefficients were useful for investigating whether any associations existed. We kept the statistical analyses to be as simple as possible due to data limitations. The collinearity observed between land use and low SES, especially in Edmonton, suggests the participation of more complex variable interactions. More sophisticated calculations may be performed in the future to explore interactions with other environmental variables. For a more epidemiological approach, future research may use rates,^25,46^ if the heath databases are amenable.

The observational study design precluded any casual relationships, but instead identified differences on where hot spot patterns corresponded in space and time for birth outcomes in the 2 main cities of Alberta.

For this analysis, we prepared a static postal code file spanning beyond the minimum and maximum years of the study. This was necessary because growing communities received more postal delivery routes over time, so that later births were counted in the same spatial location as earlier births.

Instead of blindly assigning land use and SES values at the centroid, spatial inaccuracy was minimized by measuring the proportions of land use and SES categories surrounding the focal hot spot hexagons. The hexagon size was subject to the modifiable areal unit problem.^47^ Although the positioning of the hexagon grid may not be optimal for all areas of each CMA, the 1-km dimension was found by experimentation to be appropriate for urban neighborhood analysis. And as mentioned above, hexagons have less edge effects than squares and more closely match the circular neighborhood used in focal statistics.^36^

The user-friendly space-time cube tools allowed for rapid visualization and quantification of areas with statistically significant increasing or decreasing trends of SGA/LBWT. The choice of spatial and temporal aggregation can be changed to address different research questions that may inform policy decisions on where to focus on monitoring or mitigating potential risk factors at the identified hot spots.

We were able to map the spatiotemporal trends of babies born too small, which had the end result of 2-dimensional maps for the entire time period. Then, we took the analysis to the next level by associating those patterns with the surrounding environment to discover potential processes.

## Conclusions

The mapping of spatial-temporal hot spots indicated that ciSGA/ciLBWT admitted to NICUs occurred in different areas than all SGA/LBWT—not what would be expected, which was that the critically ill would occur randomly, but there were space-time hot spots indicating they were not and there was low correlation with hot spots for all. The dominant area-level associations with all SGA and LBWT hot spot patterns were primarily higher proportions of surrounding low SES and industrial land use, directly answering our research objective to help understand why the patterns were different. Less has been known about the space-time distributions and environmental association of the critically ill. In this study, we identified that only surrounding land use was associated with ciLBWT. However, industrial land use or SES was not related to the ciSGA hot spots, suggesting that different mechanisms may be in place and indicating that further research is warranted on including environmental exposures (such as air pollution from traffic and industrial sources) and maternal factors in the hot spot analyses. Space-time cubes and emerging hot spot analyses promise to be useful for any public health investigation in space and time. This is the first known study examining spatial-temporal hot spots of all and critically ill SGA/LBWT.

## Supplemental Material

EHI_Supplemental_2019_xyz23690ca15f88b – Supplemental material for Spatiotemporal Patterns of Small for Gestational Age and Low Birth Weight Births and Associations With Land Use and Socioeconomic Status Click here for additional data file. Supplemental material, EHI_Supplemental_2019_xyz23690ca15f88b for Spatiotemporal Patterns of Small for Gestational Age and Low Birth Weight Births and Associations With Land Use and Socioeconomic Status by Charlene C Nielsen, Carl G Amrhein, Prakesh S Shah, Khalid Aziz and Alvaro R Osornio-Vargas in Environmental Health Insights
