# Supplementary material for: Spatiotemporal Patterns of Small for Gestational Age and Low Birth Weight Births and Associations With Land Use and Socioeconomic Status
Source: Environ Health Insights. 2019 Aug 22;13:1178630219869922. doi: 10.1177/1178630219869922 (PMC6709433; doi:10.1177/1178630219869922)
Supplement: EHI_Supplemental_2019_xyz23690ca15f88b – Supplemental material for Spatiotemporal Patterns of Small for Gestational Age and Low Birth Weight Births and Associations With Land Use and Socioeconomic Status [file EHI_Supplemental_2019_xyz23690ca15f88b.pdf]

Supplemental Material

Spatiotemporal patterns of small for gestational age and low birth weight births and associations with land use and socioeconomic status

Charlene C. Nielsen[1,2], Carl G. Amrhein[2,3], Prakesh S. Shah[4], Khalid Aziz[1], and Alvaro R. Osornio-Vargas[1]

[1] Department of Pediatrics, University of Alberta, Edmonton, Canada

[2] Department of Earth and Atmospheric Sciences, University of Alberta, Edmonton, Canada

3 Faculty of Arts and Sciences, Aga Khan University, Nairobi, Kenya, and Karachi, Pakistan

[4] Department of Pediatrics and Institute of Health Policy, Management, and Evaluation, University of Toronto, Mount Sinai Hospital, Canadian Neonatal Network, Toronto, Ontario

Corresponding author info: Dr. Alvaro R. Osornio-Vargas, Department of Pediatrics, University of Alberta, 3-591 ECHA, 11405 87th Avenue, Edmonton, Alberta, Canada, T6G 1C9, osornio@ualberta.ca

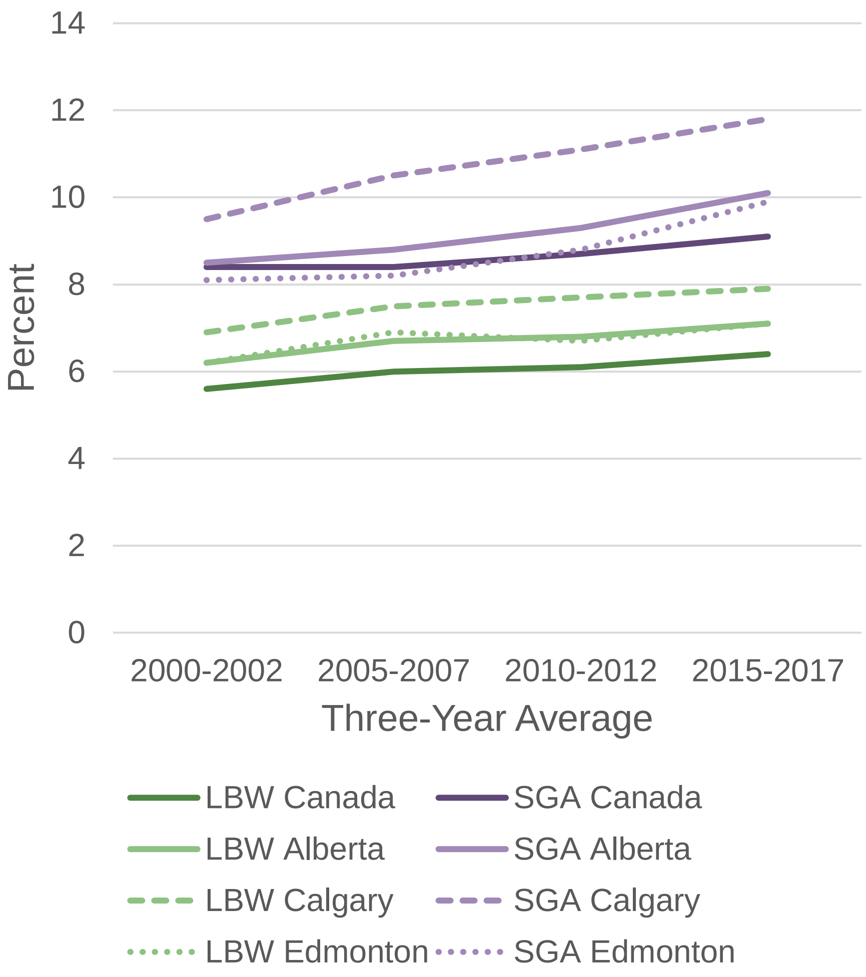

Figure S1. In Canada, the average rate of SGA was reported to be 9.1% and LBW (all gestational ages <2,500 g) was 6.4%, during 2015-2017 5; whereas in Alberta, the rate of SGA was 10.1% and LBW was 7.1%. These values have been increasing since before the beginning of our 2006-2010 study involving the Census Metropolitan Areas (CMAs) of Calgary and Edmonton.

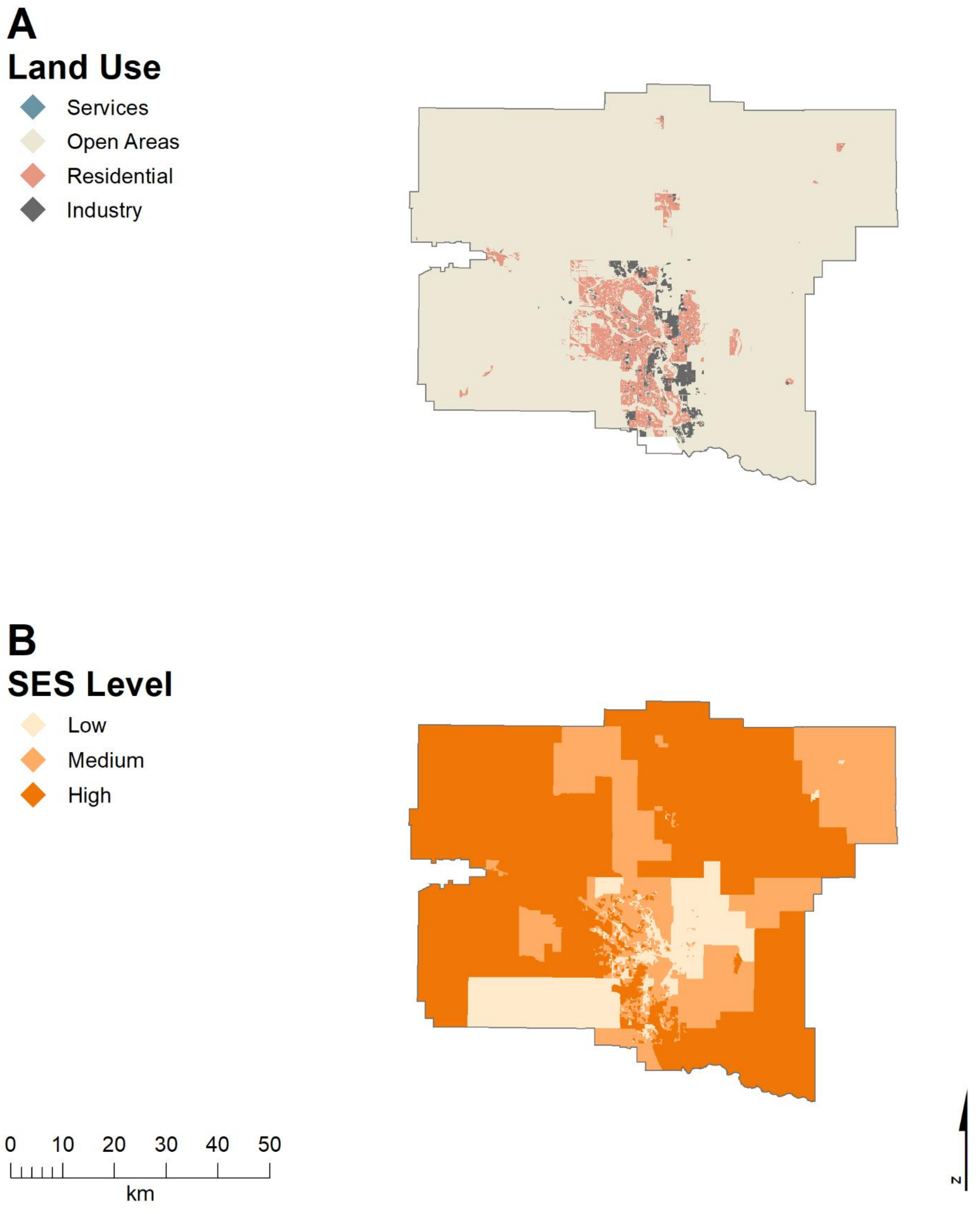

Figure S2. Calgary Census Metropolitan Area (CMA) maps for (A) land use and (B) socioeconomic status (SES).

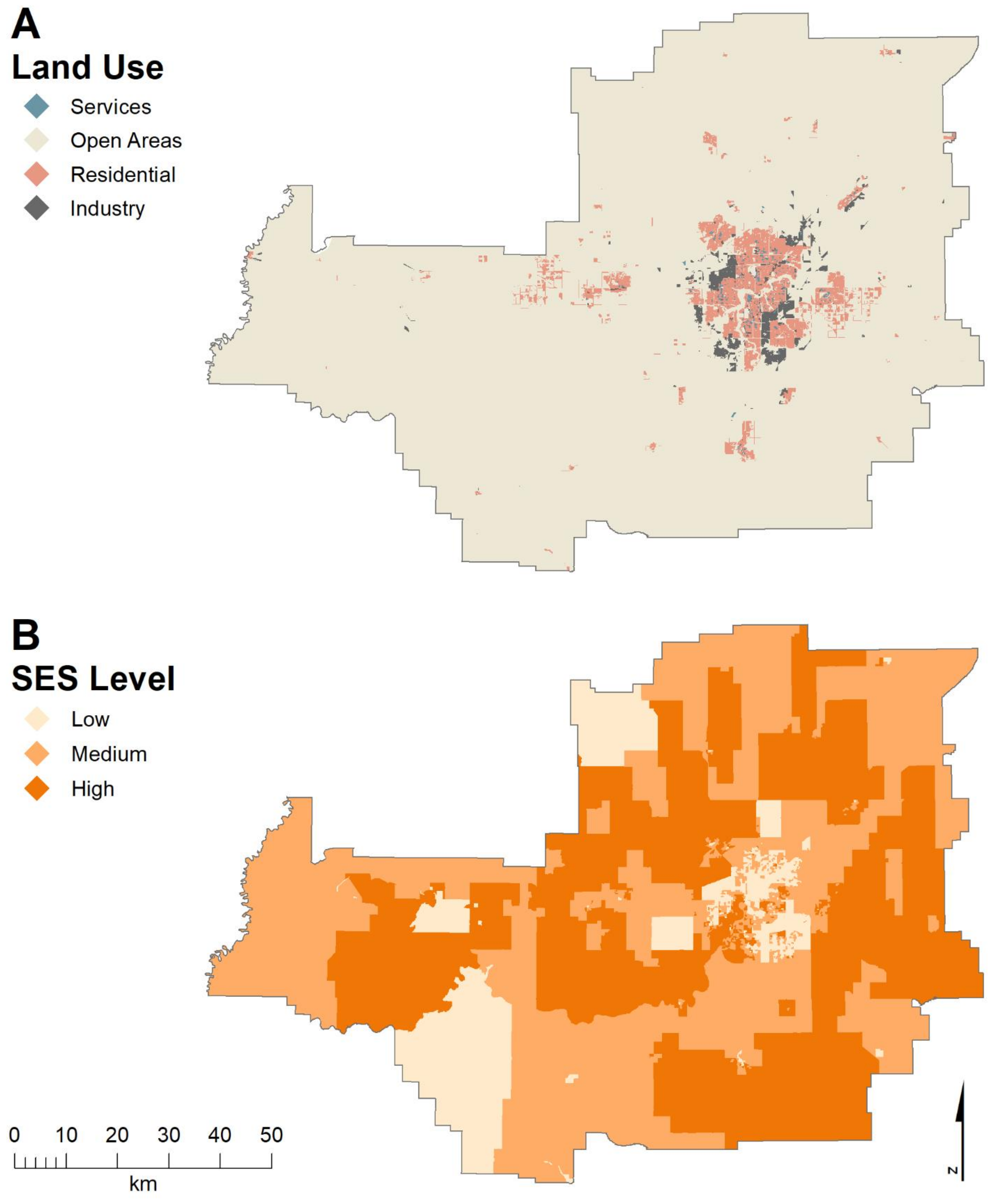

Figure S3. Edmonton Census Metropolitan Area (CMA) maps for (A) land use and (B) socioeconomic status (SES).

In Calgary there were six distinct areas of hot spot patterns for all births (indicated by red toned symbols in Figure S4). The largest patch was in the northeast, and smaller ones in the northwest, northcentral, central, southcentral, and southeast. The five distinct areas of SGA occurred in the northeastern (largest), northcentral, central, southcentral, and southeast (Figure S5). Much smaller areas were observed for ciSGA: central and scattered in the northwest (Figure S6). Figure S7 shows two separate hot spot patterns for LBWT in the northeast, one in the east, one central, and an outlying community. The distinct areas for ciLBWT were northeast, central (but expanded beyond LBWT), and in the southeast (Figure S8).

Note that Figures S9 through S13 are enlargements of Figures 4 through 6 of the main publication.

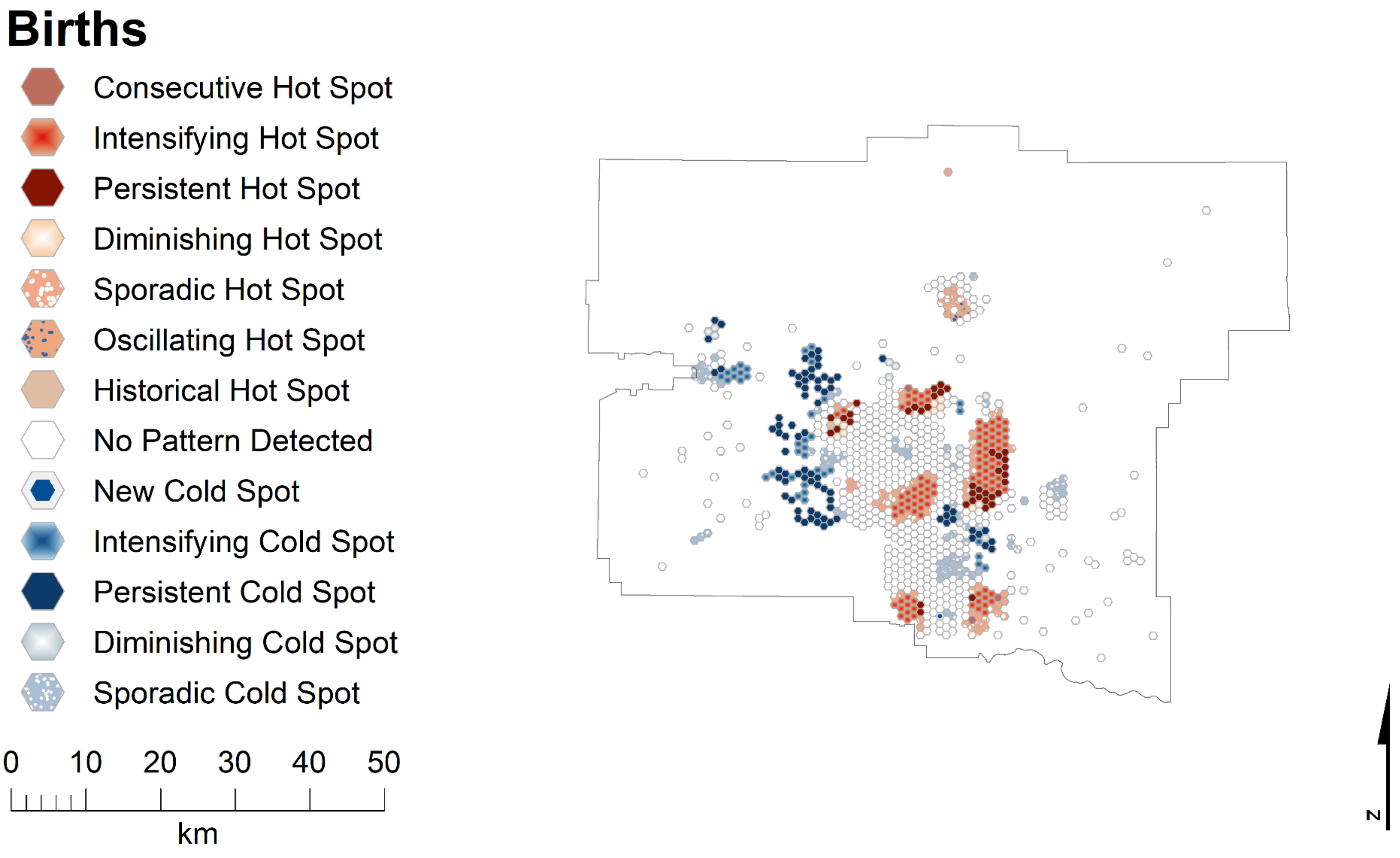

Figure S4. Emerging hot spots of all births in the Calgary CMA.

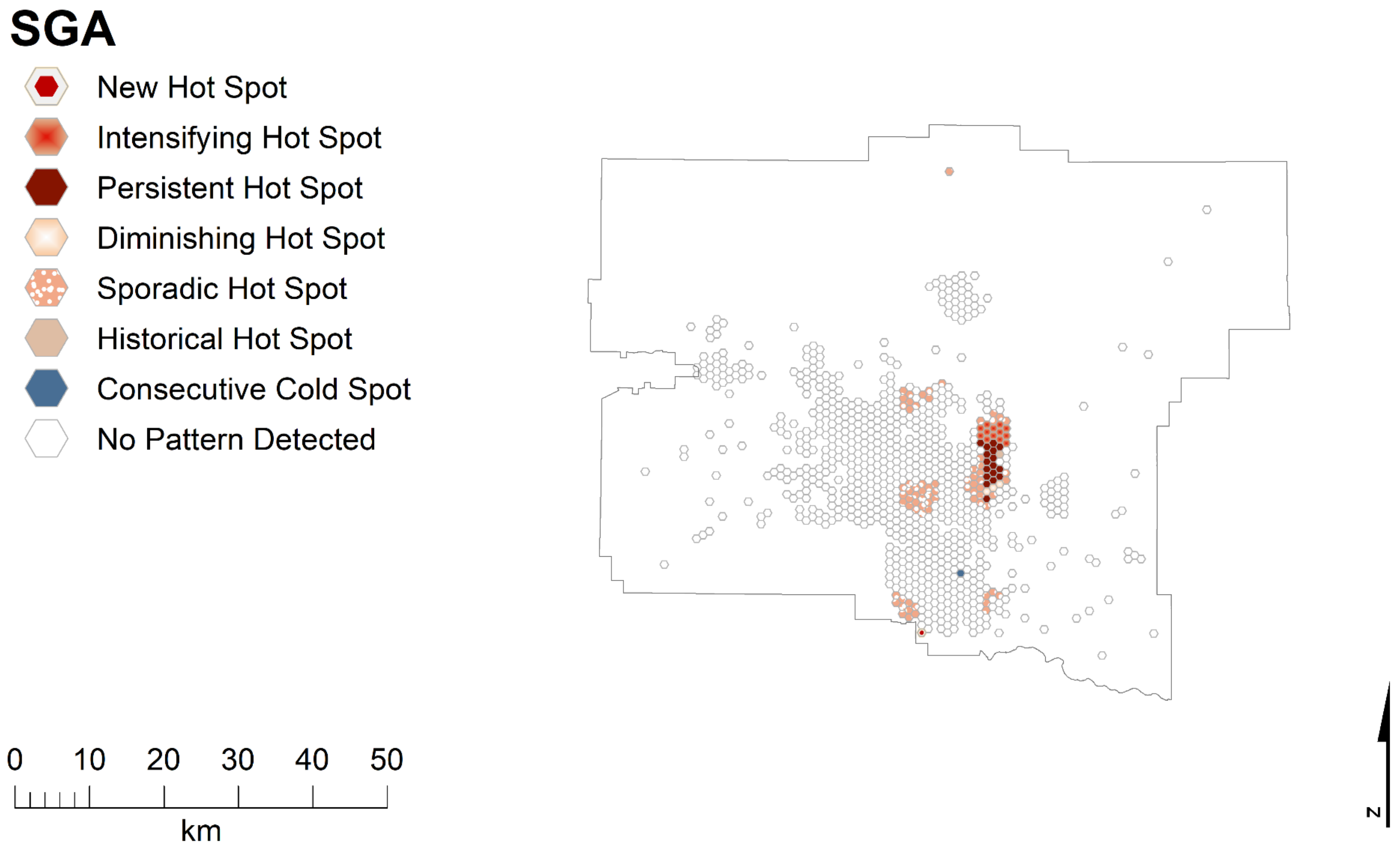

Figure S5. Emerging hot spots of all small for gestational age (SGA) in the Calgary CMA.

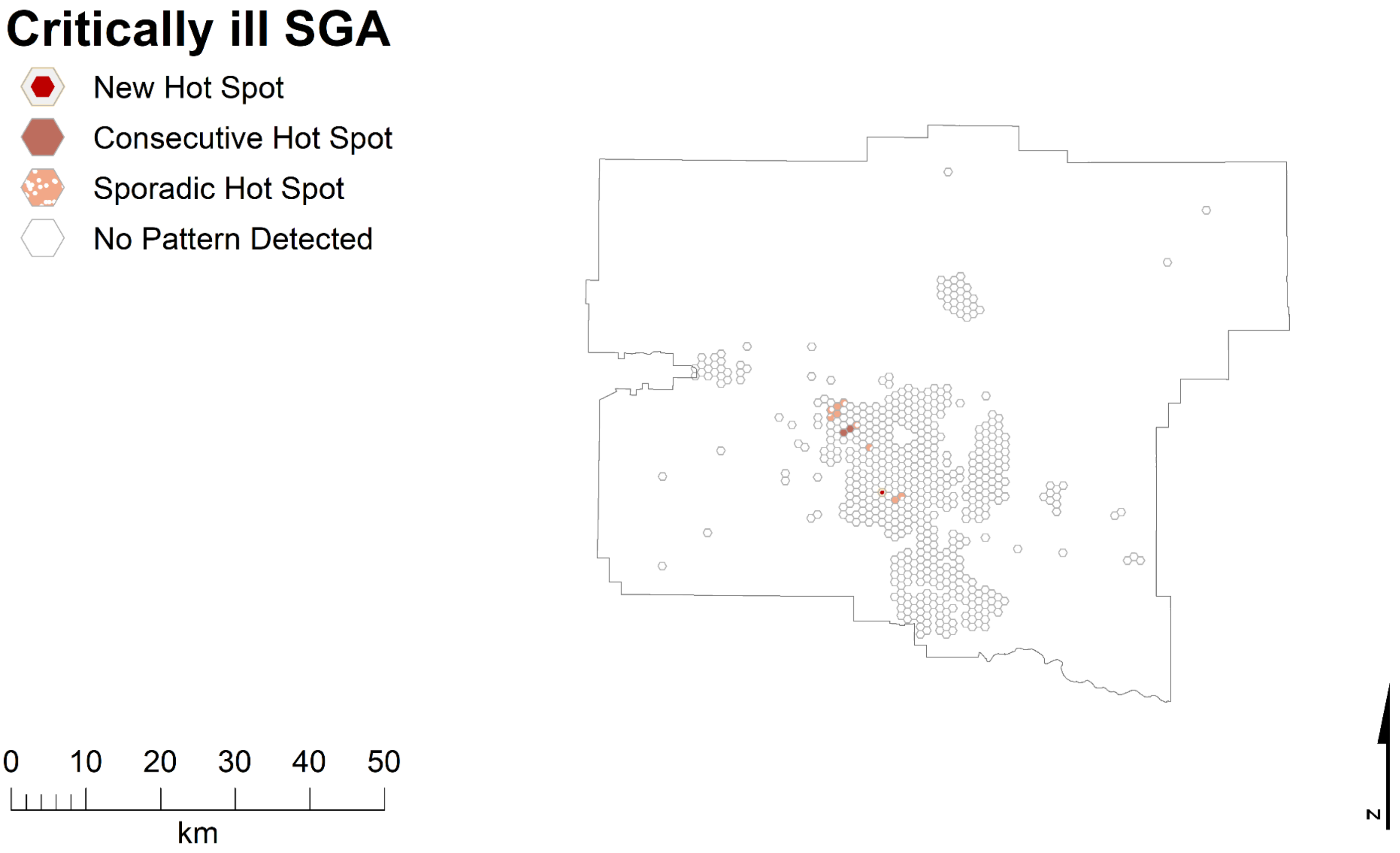

Figure S6. Emerging hot spots of critically ill small for gestational age (ciSGA) in the Calgary CMA.

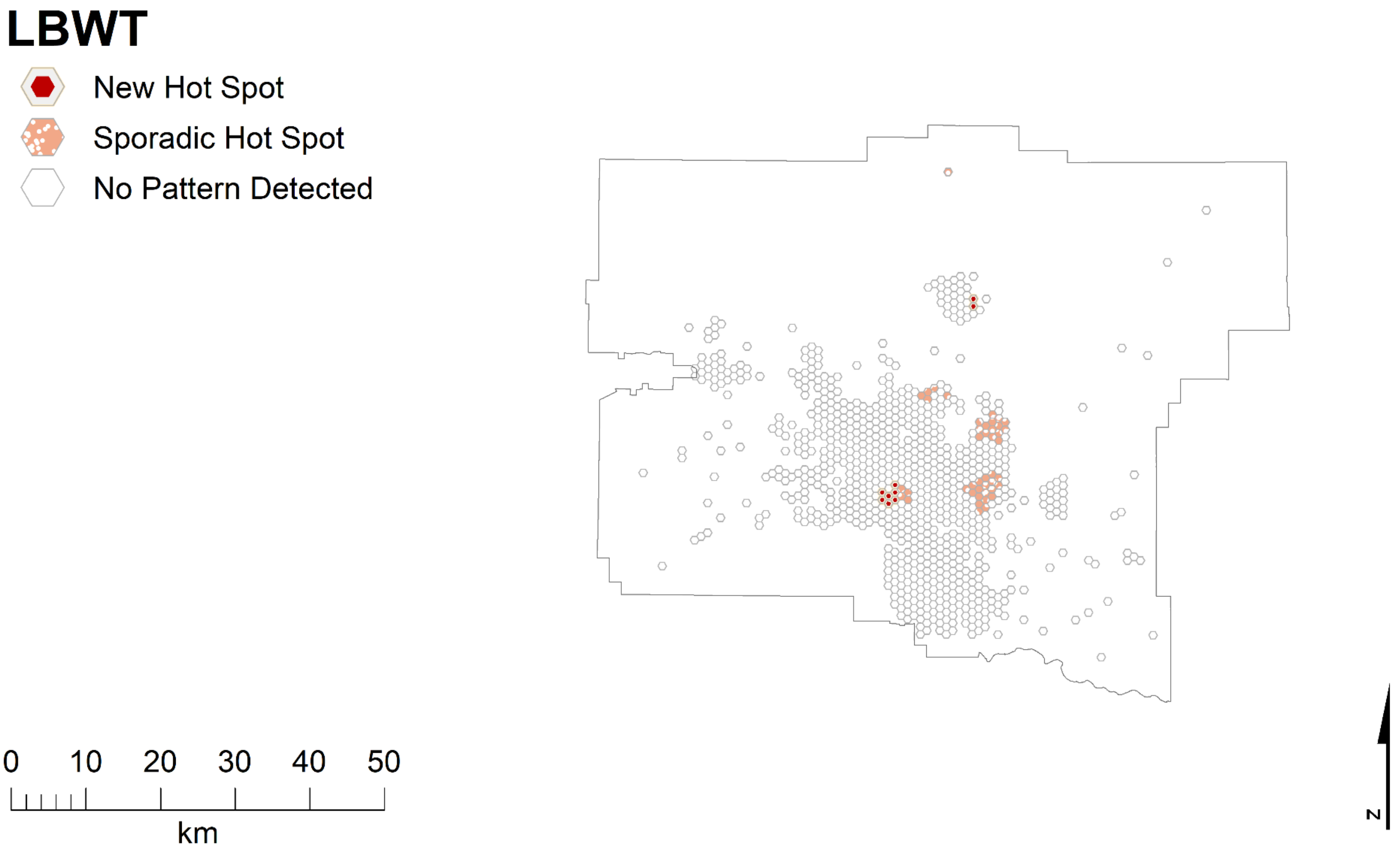

Figure S7. Emerging hot spots of all low birth weight at term (LBWT) in the Calgary CMA.

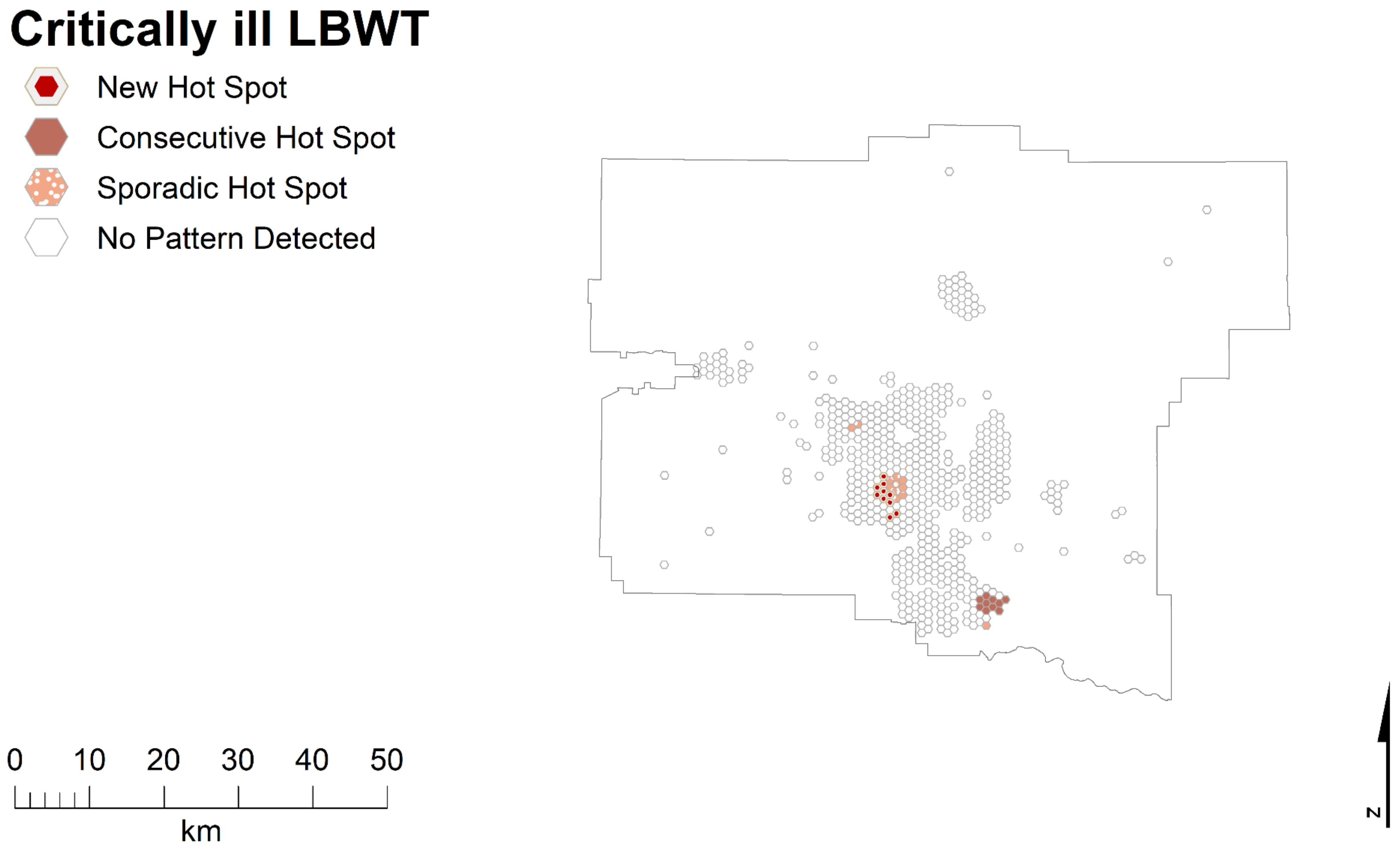

Figure S8. Emerging hot spots of critically ill low birth weight at term (ciLBWT) in the Calgary CMA.

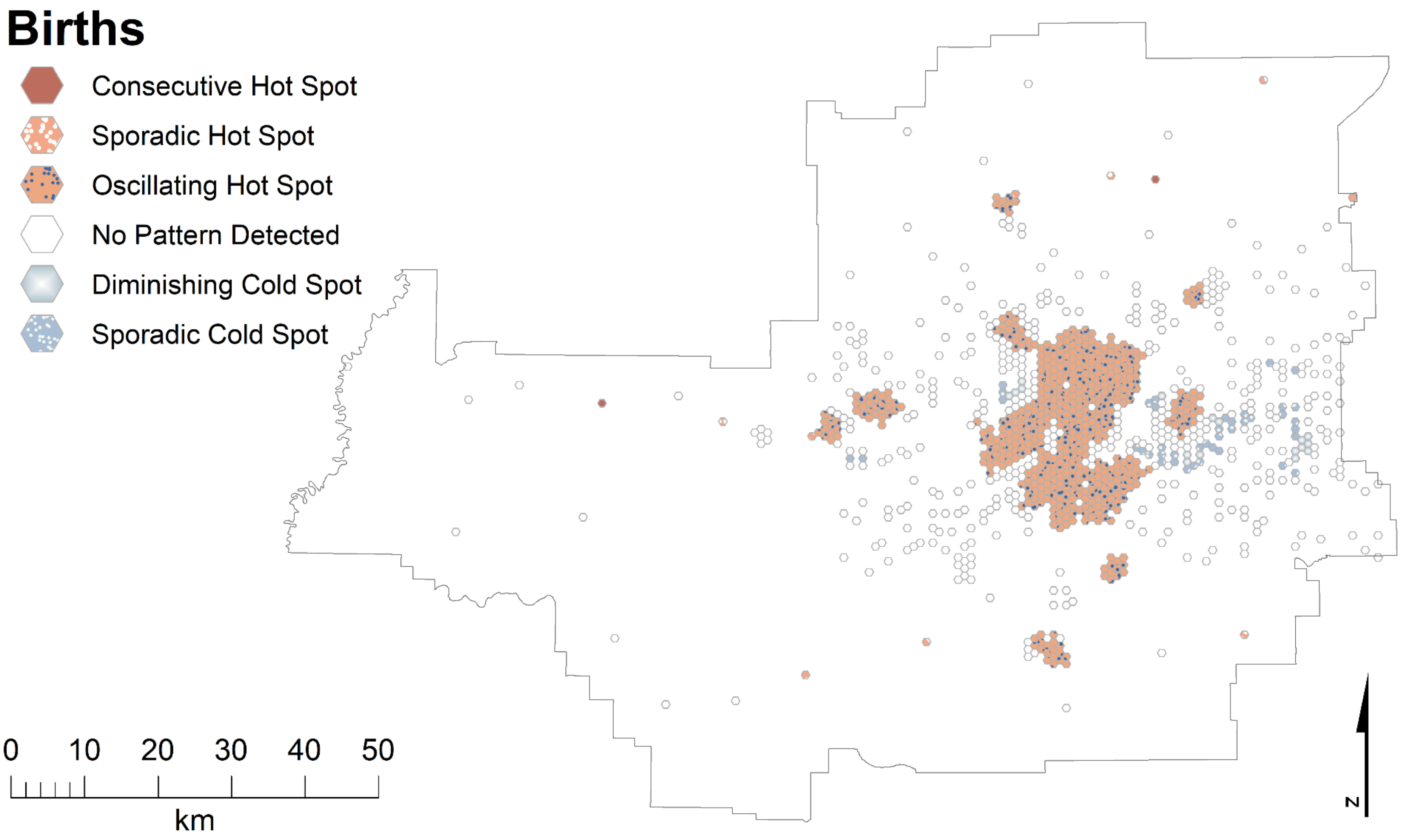

Figure S9. Emerging hot spots of all births in the Edmonton CMA.

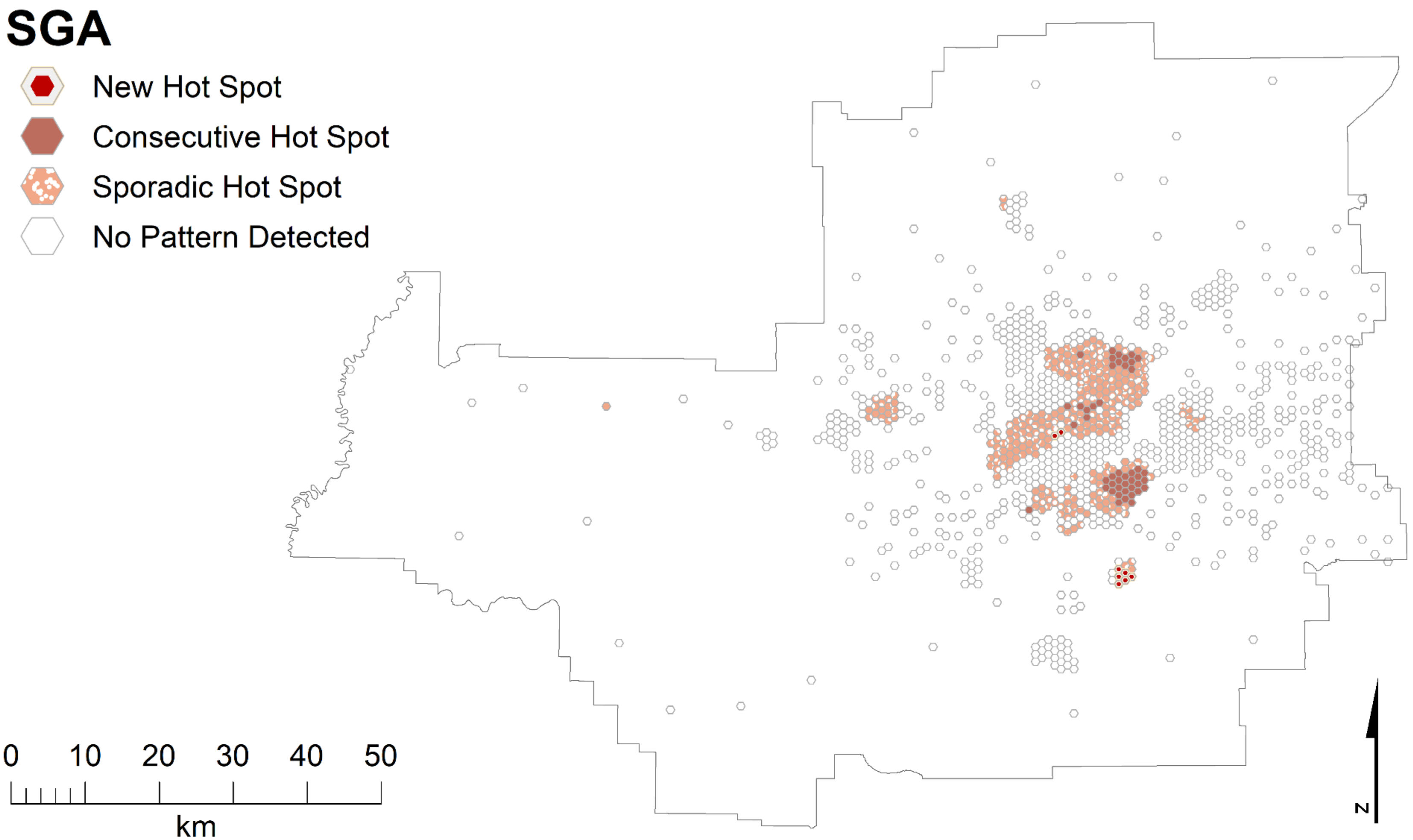

Figure S10. Emerging hot spots of all small for gestational age (SGA) in the Edmonton CMA.

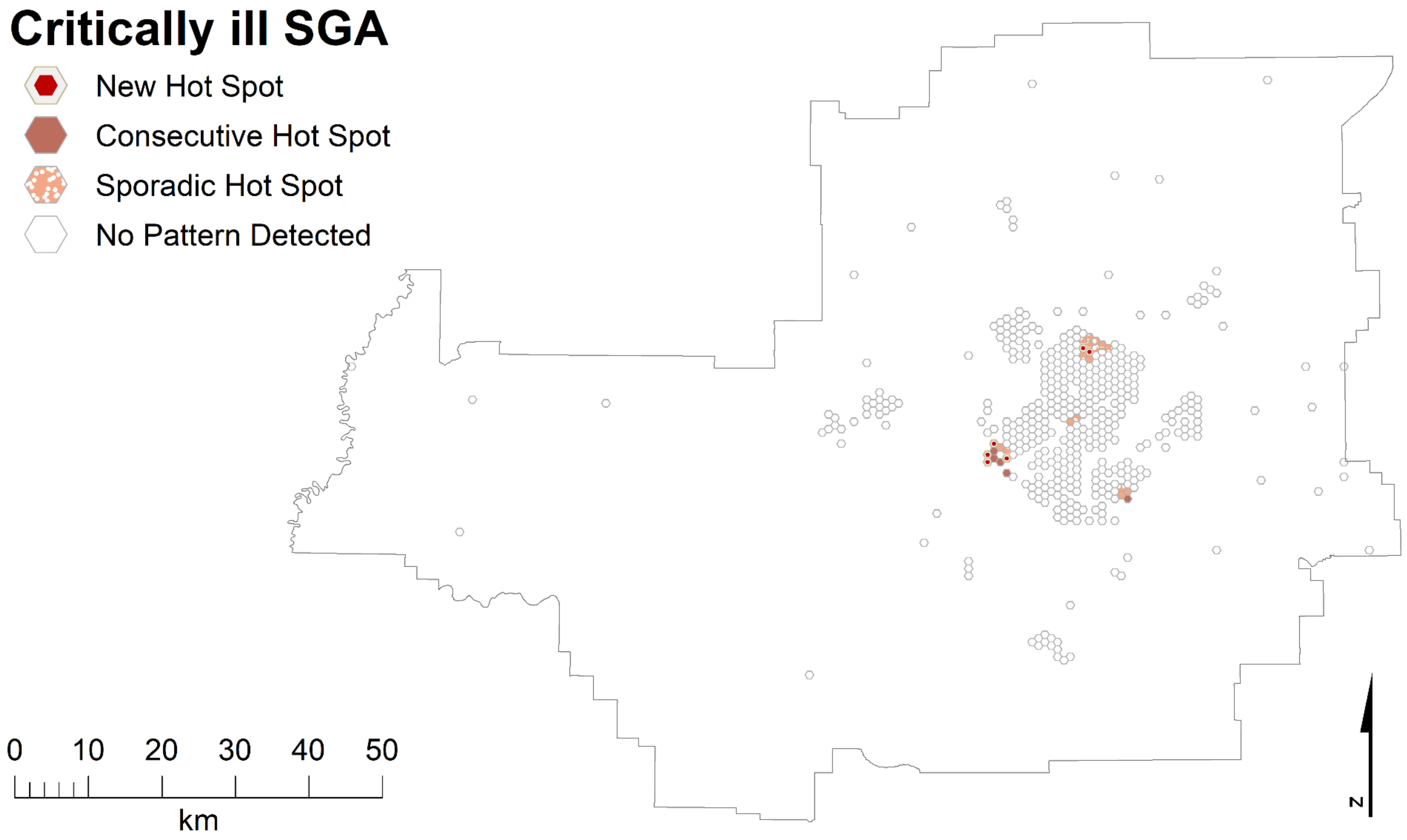

Figure S11. Emerging hot spots of critically ill small for gestational age (ciSGA) in the Edmonton CMA.

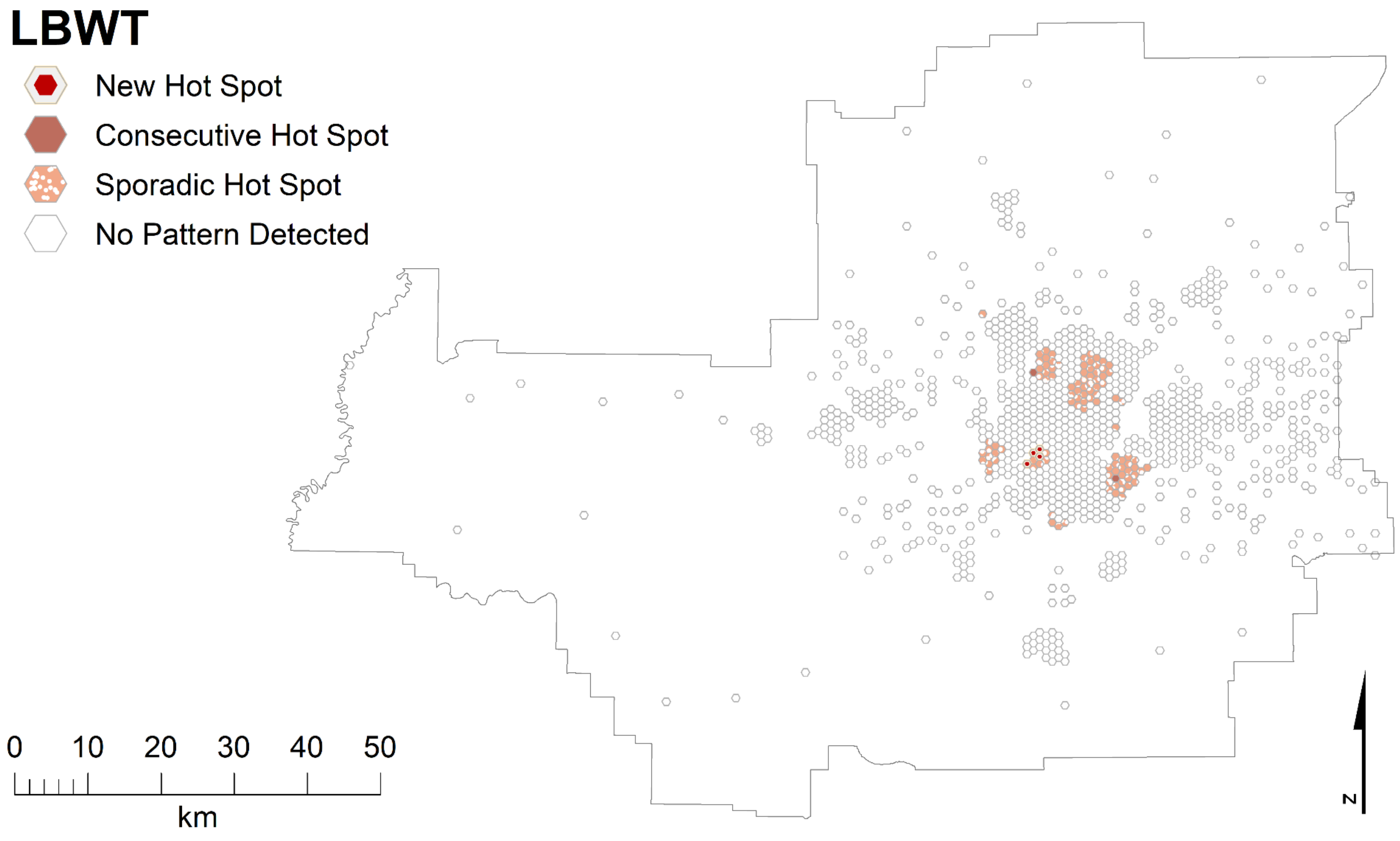

Figure S12. Emerging hot spots of all low birth weight at term (LBWT) in the Edmonton CMA.

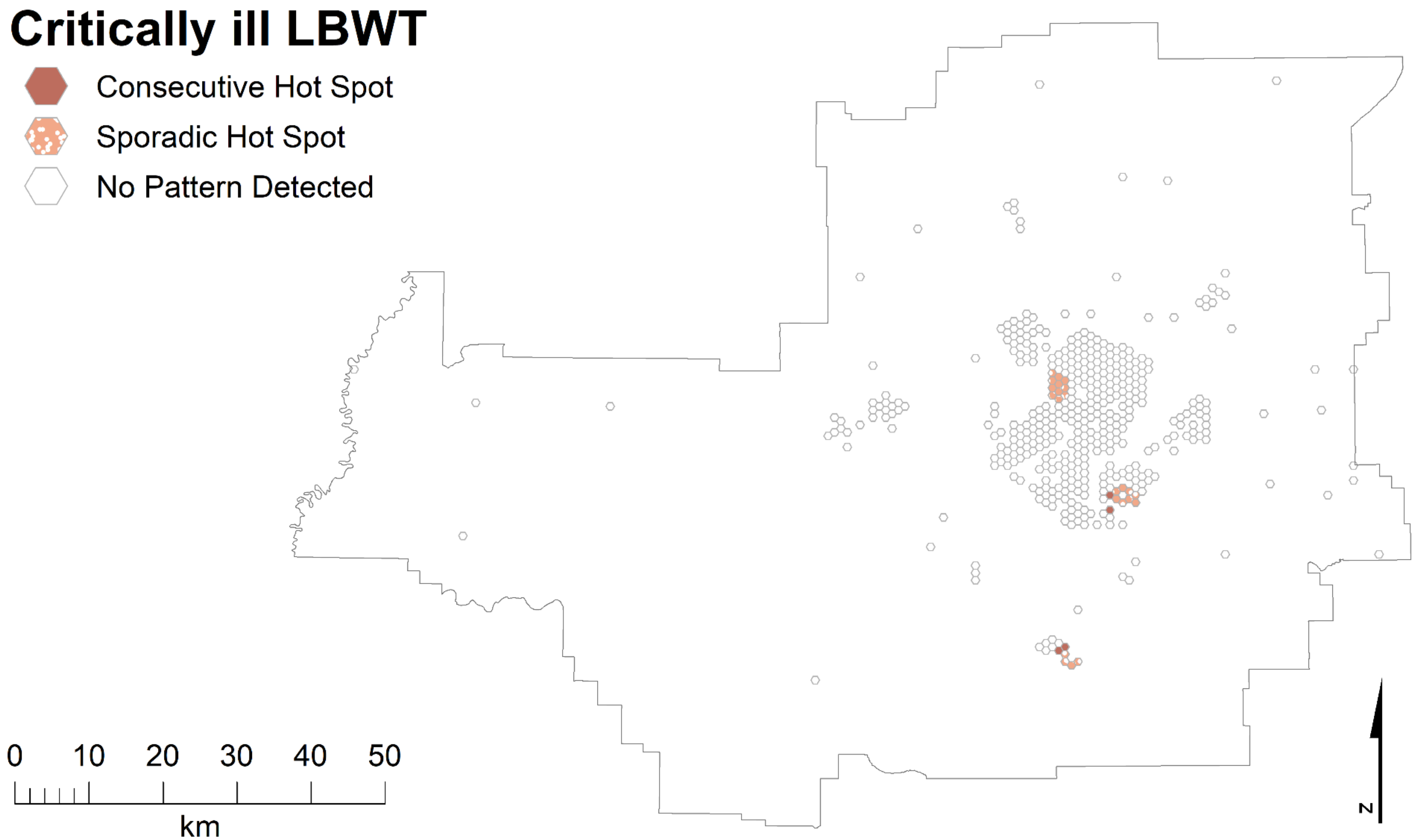

Figure S13. Emerging hot spots of critically ill low birth weight at term (ciLBWT) in the Edmonton CMA.

Table S1. Statistically significant hot spot categories for the 5- and 3-year study periods are defined in terms of the total months aggregated by 3-month time steps. Table continues.

Pattern Category	Emerging Hot Spot Definition
New Hot Spot	A hot spot location for the last 3 months of the time series (the final time-step interval) and has never been a hot spot before.
Consecutive Hot Spot	A *never-been-hot-before* location with a single uninterrupted run of hot spot bins in the final time-step intervals, and for <90% of time-step intervals (Calgary: <54 months; Edmonton <32.4 months).
Intensifying Hot Spot	A hot spot location for ≥90% of the time-step intervals (Calgary: 54 of the 60 months; Edmonton: 32.4 of the 36 months), including the last 3 months (final time step), and there is an *increase* in the intensity of clustering of *high* counts in each 3-month time step.
Persistent Hot Spot	A hot spot location for ≥90% of the time-step intervals (Calgary: 54 of the 60 months; Edmonton: 32.4 of the 36 months) and has no increasing/decreasing trend in the intensity of clustering over time.
Diminishing Hot Spot	A hot spot location for ≥90% of the time-step intervals (Calgary: 54 of the 60 months; Edmonton: 32.4 of the 36 months), including the last 3 months (final time step), and there is a *decrease* in the intensity of clustering of *high* counts in each 3-month time step.
Sporadic Hot Spot	A hot spot location that is *on-again then off-again* for <90% of time-step intervals (Calgary: <54 months; Edmonton <32.4 months), and none of the time-step intervals have been cold spots.
Oscillating Hot Spot	A hot spot location for the last 3 months (the final time-step interval) that has previously been a cold spot, and <90% of time-step intervals (Calgary: <54 months; Edmonton <32.4 months) have been hot spots.
Historical Hot Spot	A location that is *not* a hot spot for the last 3 months (the final time-step interval), but ≥90% of the time-step intervals (Calgary: 54 of the 60 months; Edmonton: 32.4 of the 36 months) have been hot spots.
No Pattern Detected	Does not fall into any of the hot or cold spot patterns defined above or below.
New Cold Spot	A cold spot location for the last 3 months of the time series (the final time-step interval) and has never been a cold spot before.
Consecutive Cold Spot	A *never-been-cold-before* location with a single uninterrupted run of cold spot bins in the final time-step intervals, and <90% of time-step intervals (Calgary: <54 months; Edmonton <32.4 months).

Pattern Category	Emerging Hot Spot Definition
Intensifying Cold Spot	A cold spot location for ≥90% of the time-step intervals (Calgary: 54 of the 60 months; Edmonton: 32.4 of the 36 months), including the last 3 months (final time step), and there is an *increase* in the intensity of clustering of *low* counts in each 3-month time step.
Persistent Cold Spot	A cold spot location for ≥90% of the time-step intervals (Calgary: 54 of the 60 months; Edmonton: 32.4 of the 36 months) and has no increasing/decreasing trend in the intensity of clustering over time.
Diminishing Cold Spot	A cold spot location for ≥90% of the time-step intervals (Calgary: 54 of the 60 months; Edmonton: 32.4 of the 36 months), including the last 3 months (final time step), and there is a *decrease* in the intensity of clustering of *low* counts in each 3-month time step.
Sporadic Cold Spot	A cold spot location that is *on-again then off-again* for <90% of time-step intervals (Calgary: <54 months; Edmonton <32.4 months), and none of the time-step intervals have been hot spots.
Oscillating Cold Spot	A cold spot location for the last 3 months (the final time-step interval) that has previously been a hot spot, and <90% of time-step intervals (Calgary: <54 months; Edmonton <32.4 months) have been cold spots.
Historical Cold Spot	A location that is *not* a cold spot for the last 3 months (the final time-step interval), but ≥90% of the time-step intervals (Calgary: 54 of the 60 months; Edmonton: 32.4 of the 36 months) have been cold spots.

Table S2. Spearman's correlation (*rho*) statistics compare emerging hot spot patterns for all births, all small for gestational age (SGA) or low birth weight at term (LBWT), and critically ill (ci) with proportions of each land use and SES category. Significant *rho* values (p<0.05) are marked with an asterisk (*).

**Edmonton**					
Spearman's *rho*	Births	SGA	LBWT	ciSGA	ciLBWT
**Land Use**					
Services	0.33*	0.22*	0.00	-0.10*	0.00
Open Areas	-0.52*	-0.40*	-0.24*	-0.03	-0.09
Residential	0.48*	0.44*	0.26*	0.06	-0.03
Industry	0.35*	0.23*	0.21*	0.07	0.17*
**Socioeconomic Status**					
SES Low	0.41*	0.43*	0.38*	0.07	0.13*
SES Medium	0.13*	0.06	-0.14*	0.18*	0.05
SES High	-0.38*	-0.41*	-0.27*	-0.15*	-0.12*

**Calgary**					
Spearman's *rho*	Births	SGA	LBWT	ciSGA	ciLBWT
**Land Use**					
Services	0.04	0.14*	0.11*	-0.06	0.02
Open Areas	-0.29*	-0.23*	-0.08	-0.02	-0.17*
Residential	0.20*	0.11*	0.07	0.08*	0.11*
Industry	0.28*	0.20*	0.05	-0.07	0.12*
**Socioeconomic Status**					
SES Low	0.16*	0.38*	0.30*	-0.07	-0.07
SES Medium	-0.07	-0.13*	-0.18*	0.01	0.00
SES High	-0.23*	-0.42*	-0.30*	0.07	0.11*

Table S3. Spearman's correlation matrix of the regression model covariates: proportions of land use and socioeconomic status categories. Significant rho values (p<0.05) are marked with an asterisk (*).

Edmonton

Spearman's *rho*	SES Low	SES Medium	SES High	Services	Open Areas	Residential	Industry
SES Low	1						
SES Medium	-0.24*	1					
SES High	-0.70*	-0.34*	1				
Services	0.57*	-0.20*	-0.24*	1			
Open Areas	-0.77*	0.22*	0.50*	-0.73*	1		
Residential	0.61*	-0.2*	-0.37*	0.67*	-0.84*	1	
Industry	0.48*	0.04	-0.45*	0.14*	-0.46*	0.05	1

Calgary

Spearman's *rho*	SES Low	SES Medium	SES High	Services	Open Areas	Residential	Industry
SES Low	1						
SES Medium	0.00	1					
SES High	-0.78*	-0.40*	1				
Services	0.53*	0.04	-0.30*	1			
Open Areas	-0.44*	-0.24*	0.33*	-0.66*	1		
Residential	0.36*	0.13*	-0.17*	0.60*	-0.85*	1	
Industry	0.20*	0.33*	-0.35*	0.13*	-0.34*	-0.07	1

Table S4. Variance Inflation Factors (VIF) for logistic regression models using proportions of surrounding socioeconomic status (SES) and land use for both Census Metropolitan Areas (CMA).

**Edmonton**				
Variable	VIF	SQRT VIF	Tolerance	R-Squared
Services	2.48	1.57	0.4033	0.5967
Open Areas	4.19	2.05	0.2386	0.7614
Industrial	1.66	1.29	0.6026	0.3974
SES Low	2.24	1.50	0.4467	0.5533
SES Medium	1.12	1.06	0.8912	0.1088
Sum Births	1.51	1.23	0.6604	0.3396
Mean VIF	2.20			

**Calgary**				
Variable	VIF	SQRT VIF	Tolerance	R-Squared
Services	1.85	1.36	0.5401	0.4599
Open Areas	2.43	1.56	0.4108	0.5892
Industrial	1.37	1.17	0.7297	0.2703
SES Low	1.31	1.15	0.7623	0.2377
SES Medium	1.33	1.16	0.7496	0.2504
Sum Births	1.29	1.14	0.7733	0.2267
Mean VIF	1.60			

Table S5. Logistic regression β coefficients (and 95% CI) for all and critically ill SGA/LBWT, modelled with proportions of only socioeconomic status (SES) and sum of all births for each location. Calgary also included industrial land use because it was not correlated with SES (see Table S3). Significant coefficients (p<0.05) marked by an asterisk (*); number of locations are indicated in Table 3.

Edmonton

β coefficient (95% CI)	SGA	LBWT	ciSGA	ciLBWT
SES Low	4.4 (3.7, 5.0)*	4.3 (3.3, 5.3)*	-0.9 (-1.5, -0.3)*	-0.6 (-1.3, 0.0)
SES Medium	2.7 (2.0, 3.4)*	1.3 (0.2, 2.5)*	-0.3 (-0.9, 0.2)	-0.5 (-1.1, 0.0)
Sum Births	0.02 (0.01, 0.02)*	0.01 (0.00, 0.01)*	-0.04 (-0.04, -0.03)*	-0.04 (-0.05, -0.03)*
Intercept	-3.88 (-4.37, -3.4)*	-4.54 (-5.38, -3.71)*	2.12 (1.8, 2.44)*	2.29 (1.95, 2.62)*
Pseudo R^2	0.36	0.23	0.32	0.35

Calgary

β coefficient (95% CI)	SGA	LBWT	ciSGA	ciLBWT
Industrial	2.5 (0.4, 4.6)*	-3.0 (-5.9, -0.2)*	0.0 (-1.7, 1.9)	2.3 (0.4, 4.3)*
SES Low	5.0 (3.9, 6.2)*	4.0 (2.7, 5.4)*	0.5 (-0.3, 1.3)	-0.2 (-0.9, 0.6)
SES Medium	1.6 (0.1, 3.2)*	1.2 (-0.8, 3.1)	0.0 (-0.8, 0.7)	-0.7 (-1.5, -0.0)*
Sum Births	0.01 (0.01, 0.01)*	0.01 (0, 0.01)*	-0.04 (-0.05, -0.03)*	-0.02 (-0.03, -0.02)*
Intercept	-6.2 (-7.32, -5.08)*	-5.35 (-6.56, -4.14)*	1.29 (0.95, 1.64)*	1.08 (0.77, 1.39)*
Pseudo R^2	0.44	0.32	0.43	0.30
